# The Homologous Recombination Machinery Orchestrates Post-replication DNA Repair During Self-renewal of Mouse Embryonic Stem Cells

**DOI:** 10.1038/s41598-017-11951-1

**Published:** 2017-09-14

**Authors:** Eui-Hwan Choi, Seobin Yoon, Kyung-Soon Park, Keun P. Kim

**Affiliations:** 1 Department of Life Sciences, Chung-Ang University, Seoul, 156-756 Korea; 2 Department of Biomedical Science, CHA University, Seoul, Korea

## Abstract

Embryonic stem (ES) cells require homologous recombination (HR) to cope with genomic instability caused during self-renewal. Here, we report expression dynamics and localization of endogenous HR factors in DNA break repair of ES cells. In addition, we analyzed gene expression patterns of HR-related factors at the transcript level with RNA-sequencing experiments. We showed that ES cells constitutively expressed diverse HR proteins throughout the cell cycle and that HR protein expression was not significantly changed even in the DNA damaging conditions. We further analyzed that depleting Rad51 resulted in the accumulation of larger single-stranded DNA (ssDNA) gaps, but did not perturb DNA replication, indicating that ES cells were able to enter the G2-phase in the presence of unrepaired DNA gaps, consistent with the possibility that post-replication repair helps avoid stalling at the G2/M checkpoint. Interestingly, caffeine treatment inhibited the formation of Rad51 or Rad54 foci, but not the formation of γH2AX and Exo1 foci, which led to incomplete HR in ssDNA, thus increasing DNA damage sensitivity. Our results suggested that ES cells possess conserved HR-promoting machinery to ensure effective recruitment of the HR proteins to DNA breaks, thereby driving proper chromosome duplication and cell cycle progression in ES cells.

## Introduction

Blastocyst-derived ES cells are rapidly dividing pluripotent cells which have the ability to self-renewal and differentiation^1, 2^. Particularly, ES cells maintain a significantly higher level of expression of homologous recombination (HR)-related proteins compared to their expression levels in differentiated cells, leading to stable proliferation throughout the ES cell-specific cell cycle^3–5^. Thus, the cell cycle of ES cells is linked to the HR pathway, overcomes genomic instability that occurs through DNA breaks, and specifically suppresses mutations. HR is known to facilitate the efficient repair of DNA breaks, interstrand crosslinks (ICLs), and stalled replication forks. HR proteins are involved in the search for homology and strand pairing that mediate DNA strand invasion by Rad51-ssDNA presynaptic filaments to repair spontaneous DSBs. The participation of highly ordered HR machinery is required during both mitotic and meiotic cell cycles^6–8^. The HR pathway is distinct from the non-homologous end joining (NHEJ) mechanism and is restricted to the S/G2 phases of the cell cycle and certain types of DNA damage^9^. Moreover, it has been reported that mouse ES (mES) cells show a lower frequency of genomic mutations than somatic cells do^10, 11^.

In this study, we demonstrated diverse phenomena showing that mES cells favor the HR pathway to maintain cellular progression and to overcome DSB-induced cellular stress caused by long-lived ssDNA resulting from DNA damage or prolonged S-phase. First, we revealed the gene-expression patterns of numerous HR-related genes by performing RNA-Seq analysis, which showed that the HR genes involved in DNA resection, strand displacement, and resolution of joint molecules were actively expressed at similar levels in asynchronous or synchronized S-phase cultures. Although most mES cells in the asynchronous population were in the S-phase, this was not the reason that mES cells exhibited high expression of the HR proteins, as these proteins still accumulated during the G1-to-G2/M phases in synchronized mES cells. Second, we examined whether Rad51-dependent HR was essential for the fidelity and efficacy of cellular progression at the G2/M transition. During ES cell cycle, abundant HR factors may facilitate continuous DNA replication and prevent the accumulation of DNA lesions via post-replication repair, including ssDNA gaps in late S phase, and ES cells utilize the HR pathway to support genomic integrity and cell proliferation^7, 12–16^. Thus, the absence of Rad51-dependent HR might arrest ES cells at the late S-phase or G2/M phase and inhibit cell proliferation. Third, upon reducing serum concentration in the media, mES cells stalled at the G2/M phase and exhibited reduced HR protein expression and decreased cell growth rates. Fourth, the expression levels of HR proteins in mES cells following treatment with DNA damage-inducing agents were similar to the corresponding levels in untreated mES cells. Finally, we analyzed the intracellular localization of HR factors in mES cells exposed to exogenous DNA-damaging agents. Rad51, Rad54, Exo1, and γH2AX formed multiple foci following treatment with all tested chemical reagents, except for caffeine^17–21^. In addition, we provided evidence that caffeine could be used to control HR-mediated DNA repair during cell cycle and proliferation of ES cells. The susceptibility of mES cells to replication stress suggests that HR pathways may affect important features of mES cells including prolonged S-phase and rapid self-renewal^15, 22–25^. In support of this idea, we reported here that an HR-dependent pathway modulated by ES cell-specific expression of HR proteins to sustain cell viability and promote proliferation could rapidly recover the delay of ES cell self-renewal caused by a large amount of ssDNA.

## Results

### mES cells express high levels of multiple factors involved in DNA-related processes including HR and DNA repair

**Figure 1:**
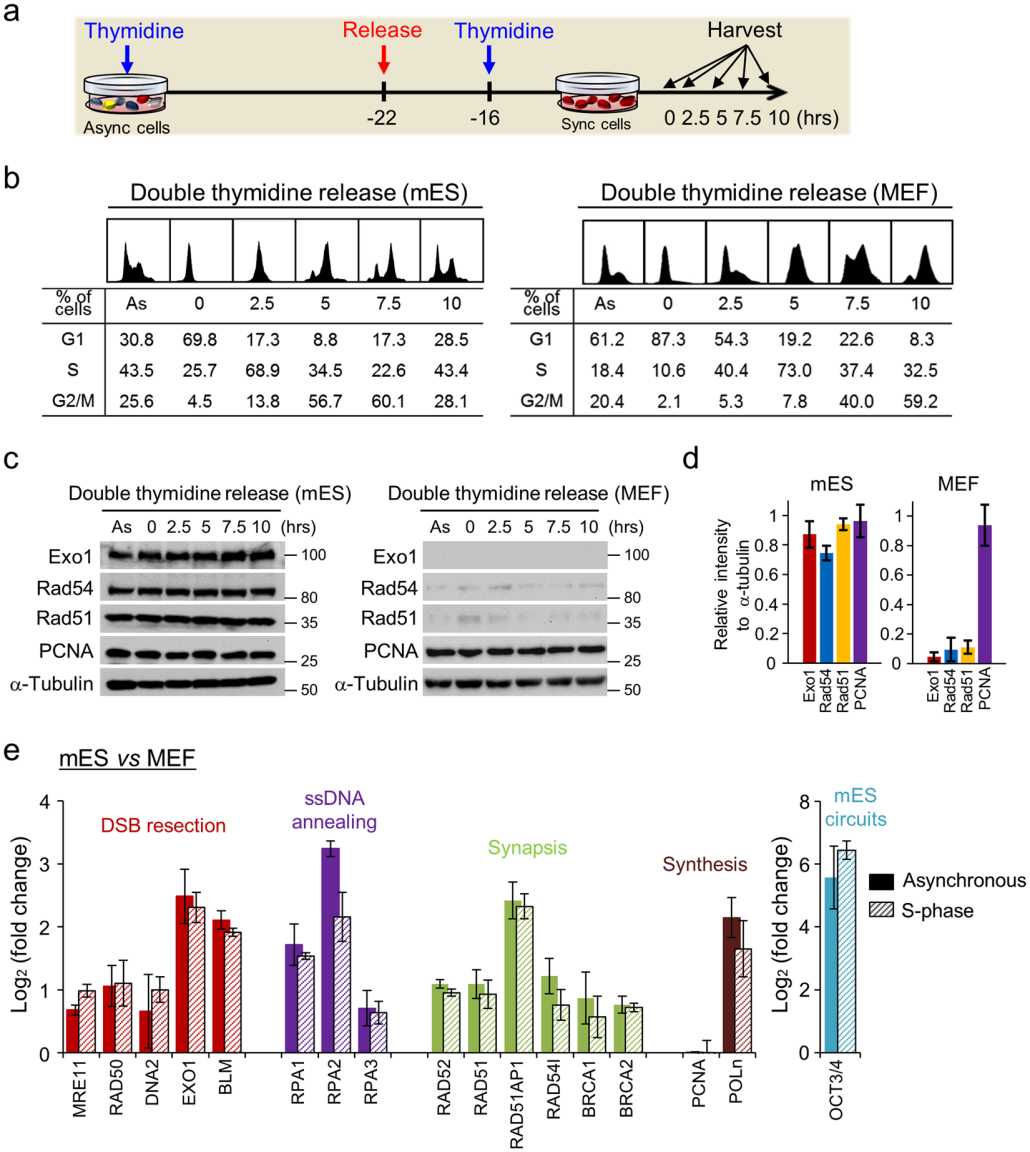
ES cells express diverse HR factors at high levels throughout cell cycle phases. (**a**) Schematic of the cell cycle time-course experiment (see Materials and Methods). mES cells were synchronized with thymidine and then released from G1/S phase. (**b**) FACS analysis showing cell cycle progression following G1/S phase synchronization in MEFs and mES cells. AS, asynchronous state. The cells were harvested at 2.5-h intervals, as indicated, and the relative proportion of cells in each cell cycle phase is shown. (**c**) Immunoblot detection of Exo1, Rad51, and Rad54 proteins after time-course experiment performed on mES cells and MEFs. α-tubulin was used as a loading control. Immunoblot signals of original membranes were detected with a ChemiDoc XRS system. DTB, double-thymidine block. (**d**) Relative HR protein expression in mES cell to HR proteins of MEFs in asynchronous cultures. Protein signals were quantified with ImageJ. (**e**) qPCR analysis for HR genes in MEFs and mES cells in asynchronous cultures or synchronized in S-phase as described in (**a**). Three independent experiments of each sample were run, and the Ct values were averaged. Log_2_ values of relative fold-changes measured in mRNA expression detected by qPCR analysis for MEFs and mES cells. Error bars indicate the mean ± SD.

We have previously reported that mES cells constitutively express high levels of Rad51 throughout the cell cycle^3^. Since Rad51-mediated HR is predominantly active in the S-to-G2 phases of the cell cycle in eukaryotic cells, we further characterized the expression patterns of diverse HR factors both in asynchronous mES cells, as well as those synchronized at the G1/S phase checkpoint using a double-thymidine block, after which the cells were released from the block and progressed through the cell cycle (Fig. 1a,b). End resection of DNA DSB, generating 3′-ssDNA, is required to facilitate HR-mediated DNA repair. Exo1 plays critical roles in the initial resection of the DSB ends, generating long ssDNA. Rad54, an accessory factor of Rad51, is also responsible for Rad51-dependent homology searching and invasion processing^26^. Standard protein analysis revealed low expression of Rad51, Rad54, and Exo1 regardless of the cycle phase in mouse embryonic fibroblasts (MEFs) (Fig. 1c), whereas mES cells showed a 5-fold increase in HR protein expression throughout the cell cycle (Fig. 1c,d). Thus, these results confirmed that mES cells constitutively expressed substantial levels of Rad51, Exo1, and Rad54 in a cell cycle-independent manner. Furthermore, real-time (RT) PCR analysis from synchronous cultures in S phase or asynchronous cultures revealed that ES cells expressed diverse key HR factors directly involved in HR-mediated DSB repair at higher levels than those in MEFs (Fig. 1e).

**Figure 2:**
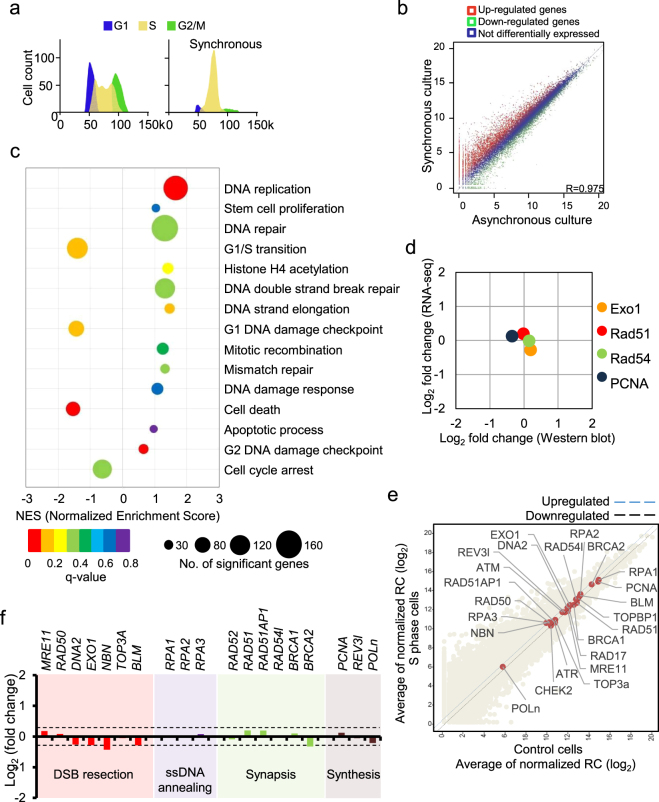
Analysis of the expression levels of HR genes in mES cells synchronized in S phase. (**a**) FACS analysis of cell cycle distribution in asynchronous and synchronous cultures. mES cells were synchronized with thymidine and then stained with DAPI to analyze DNA content. (**b**) Scatter plot of the RNA-Seq data showing upregulated or downregulated genes. (**c**) Gene set enrichment analysis (GSEA) of the cells in the asynchronous and synchronous cultures for the selected biological process gene set. NES, normalized enrichment score. (**d**) A comparison of HR gene-expression changes after synchronization in S phase, as assessed by immunoblot analysis and RNA-Seq. Log_2_ values of fold-changes measured in terms of the FPKM were compared with fold-changes in protein expression measured in asynchronous and synchronous cultures. (**e**) Scatter plot showing fold-changes in the expression of HR-related genes between asynchronous and synchronous cultures. The X and Y axes represent the average normalized read count (log_2_). (**f**) Gene sets involved in the HR pathway were classified into DSB resection, ssDNA annealing, synapsis, and synthesis. The values of the dotted line (±0.32 log_2_) represent the fold-change of β-actin expression from four-independent RNA-Seq experiments.

To identify the expression patterns of multiple genes known as HR factors and multiple genes specifically expressed from the transcript level response to the S-phase state in mES cells, we performed Gene Set Enrichment Analysis (GSEA) with RNA-Seq data (Fig. 2 and Supplementary Fig. S1). Read count values were normalized at specific stages, where cells were controlled in asynchronous culture or synchronized culture at S-phase. mES cells were synchronized in S-phase by a double-thymidine block, after which they were arrested at G1/S phase. Then, the cells were released from the arrest, allowed to progress, and harvested at S phase (Fig. 2a). We selected the gene classes that are biological process gene sets, regulated by the S-phase state, from GSEA analysis (Fig. 2b,c). The gene sets of DNA replication and G2 damage checkpoints contained hundreds of genes that were most highly upregulated in S-phase (Fig. 2c). In addition, the gene sets of DNA repair and DSB repair showed similar expression levels in both asynchronous and synchronous at S phase cultures (Fig. 2c). Furthermore, similar observations were obtained from western blot analysis and fold change analysis of RNA-Seq data (Fig. 2d). Overall, RNA-Seq results demonstrated that transcript levels of the genes involved in HR regulation exhibited no significant change in the synchronized S-phase culture. We further confirmed that S-phase cultured cells did not exhibit significantly changed transcript levels of numerous HR-specific genes involved in DSB resection, ssDNA annealing, strand invasion, and synthesis (Fig. 2e,f and Supplementary Fig. S1A). These data are consistent with the protein analysis of Rad51, Rad54, and Exo1 at G1-to-G2/M cell cycle under the same conditions, i.e., synchronizing cells with a double-thymidine block (Fig. 1). Thus, both asynchronous and synchronized cultures at S-phase tended to exhibit the same expression levels of HR-related genes, suggesting that HR-promoting signaling could be functional throughout the entire cell cycle in mES cells.

### DNA damaging-reagents do not affect the expression levels of HR proteins, but HR factors are rapidly redistributed to sites of DNA damage

**Figure 3:**
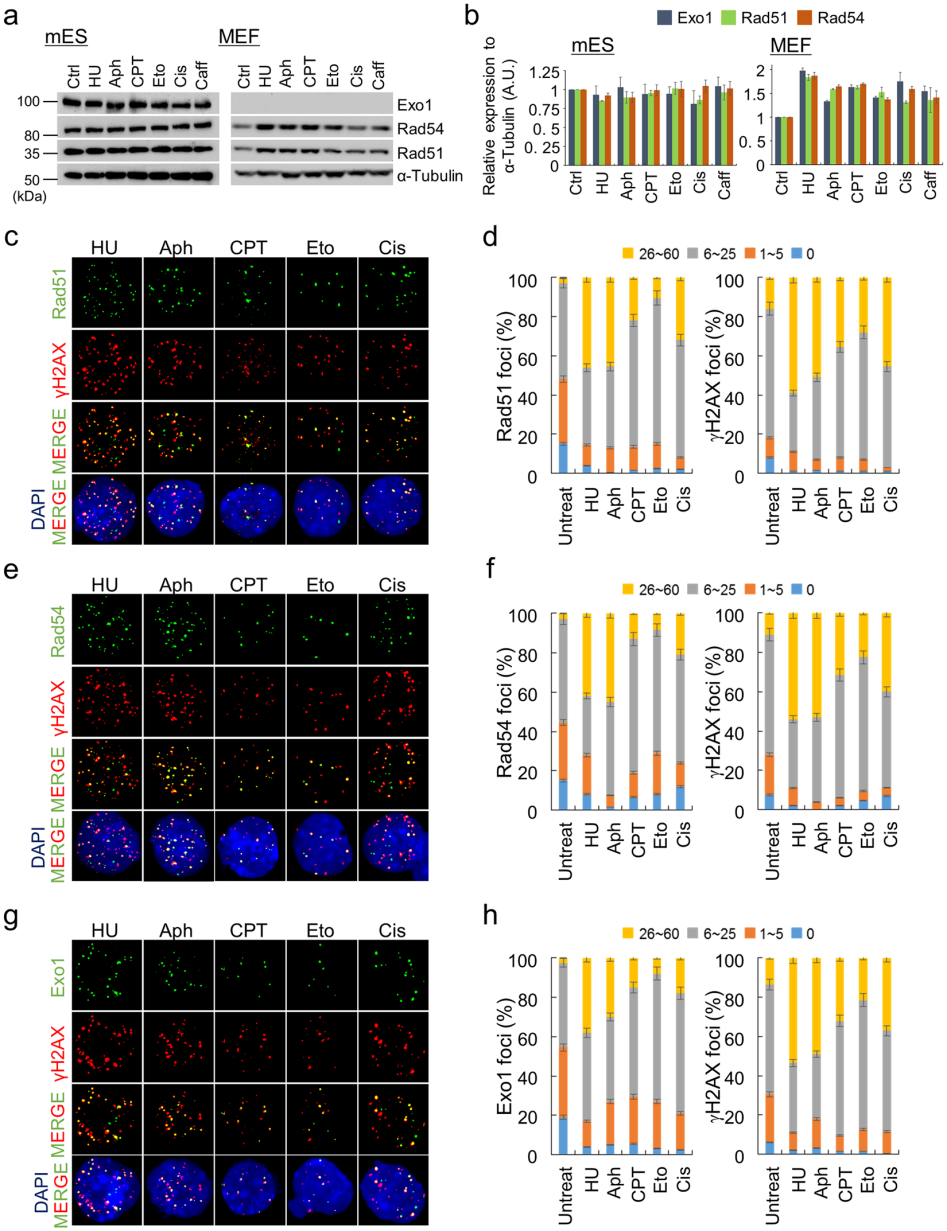
Redistribution of HR factor expression in mES cells in response to DNA damage. (**a**) Immunoblot detection of Rad51, Rad54, and Exo1 in response to various DNA damage-inducing agents. HU, hydroxyurea; Aph, aphidicolin; CPT, camptothecin; Eto, etoposide; Cis, cisplatin; Caff, caffeine). (**b**) The levels of each protein in (A) were quantified, and the ratios relative to α-tubulin were determined for each time point. Three independent experiments of each protein were performed in mES cells and MEFs on the indicated chemical reagents. Error bars indicate the mean ± SD. (**c**–**h**) Increased number of HR-foci in mES cells in response to DNA damage. mES cells were immunostained with antibodies against Rad51 and γH2AX (**c**), Rad54 and γH2AX (**e**), and Exo1 and γH2AX (**g**) after chemical treatments. (**d**,**f**,**h**) Quantification of HR foci before (untreated) and after the indicated chemical treatments. Cells displaying focal signals were categorized according to the number of foci per nucleus. More than 200 cells were counted for each experiment. Error bars indicate the mean ± SD.

Unchallenged mES cells stably express substantial levels of HR proteins throughout the cell cycle (Fig. 1c). Under DNA-damaging conditions, Rad51-mediated HR occurs to complete DNA repair, thereby maintaining genomic integrity. To investigate the response of the HR proteins Rad51, Rad54, and Exo1 in mES cells growing in an unfavorable environment, we induced DNA damage with various chemical reagents (Fig. 3). mES cells cultured in medium containing hydroxyurea (HU) and aphidicolin (Aph) failed to progress to S phase or underwent apoptosis; thus, 82% of the cells remained in G1/S-phase. Treatment with camptothecin (CPT), etoposide (Eto), and cisplatin (Cis) arrested mES cells in S/G2-phase, suggesting that these cells were unable to complete DNA replication and reach the G2 phase (Supplementary Fig. S2A). Moreover, these chemical reagents, which block DNA replication and induce DNA damage, can cause cell death via apoptosis (Supplementary Fig. S2B,C).

Although the cell cycle profiles of mES cells exposed to various DNA damage-inducing agents differed slightly, the expression levels of the HR factors did not differ significantly (Fig. 3a,b). The overall levels of HR proteins were unaffected by DNA-damage checkpoint activation because these proteins were already abundantly expressed prior to exposure to the DNA-damaging agents. However, the localization of HR proteins to DNA breaks is also particularly important for DNA repair via HR; therefore, we analyzed HR-protein focus formation in the cells after chemical treatment by immunofluorescence microscopy (Fig. 3c–h). Following treatment of mES cells with DNA break-inducing agents, Rad51, Rad54, Exo1, and γH2AX proteins redistributed and were concentrated in the nucleus as discrete foci (Fig. 3c-h). Thus, regardless of the source of DNA damage, Rad51, Rad54, and Exo1 proteins immediately colocalized with similar proportions of γH2AX foci. Taken together, we suggest that Rad51, Rad54, and Exo1 are constitutively abundant in mES cells to enable quick and efficient HR-mediated DNA repair.

### ES cells require Rad51-mediated HR to promote G2/M progression, but not DNA replication

**Figure 4:**
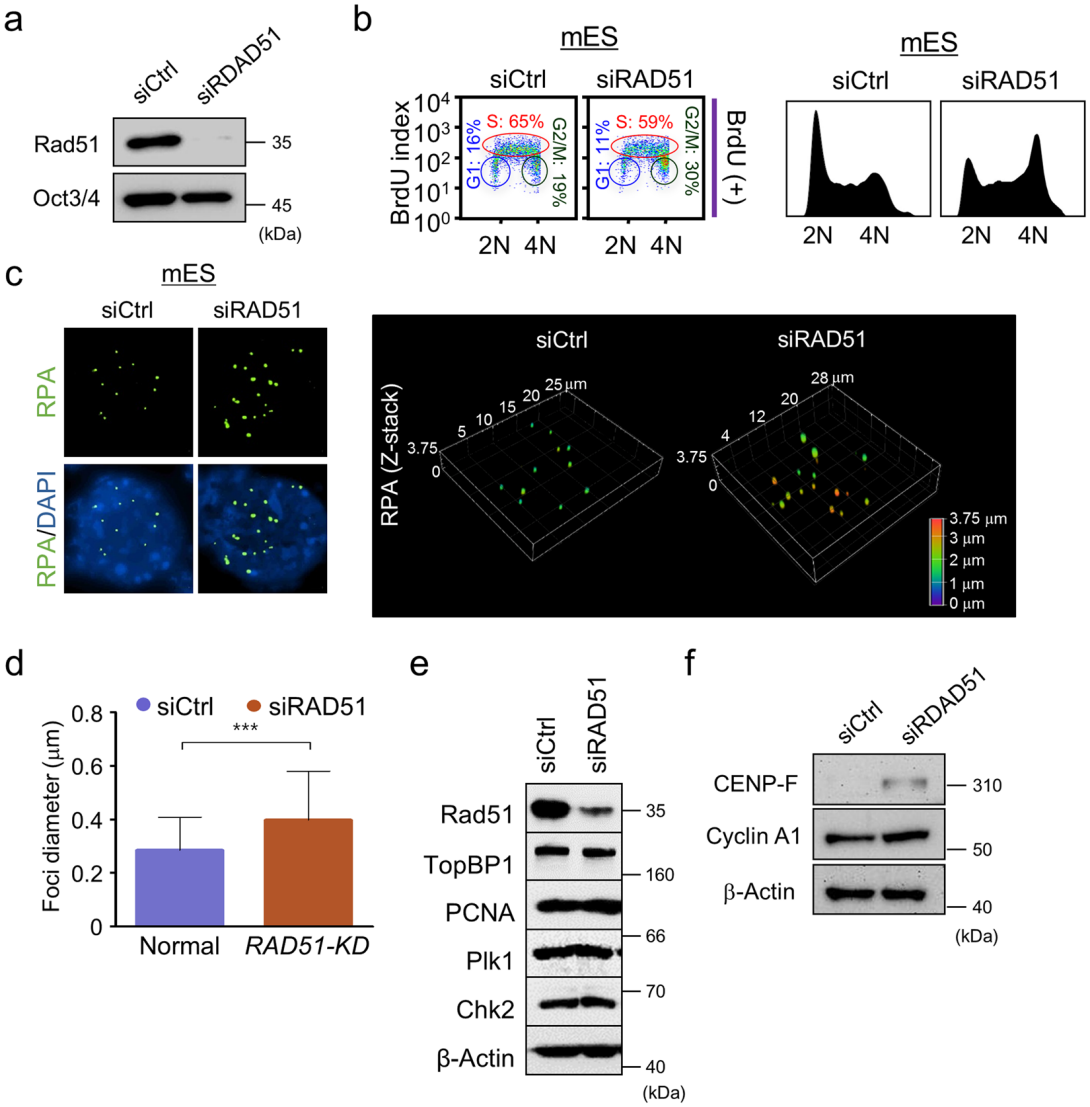
Analysis of cell cycle and protein expression in mES cells after siRAD51 treatment. (**a**) Immunoblot analysis of Rad51 and Oct3/4 proteins upon small interference RNA (siRNA) treatment. Rad51 expression in mES cells was inhibited by transfection with an siRNA pool against Rad51 (siRAD51). Cells transfected with a non-targeting siRNA (siCtrl) were also included. Oct3/4 was used as a marker of stemness. (**b**) Analysis of the cell cycle profiles after siRAD51 transfection. The BrdU index of cells was analyzed by FACS. The relative population of siRAD51-transfected mES cells in each cell cycle phase was quantified in the presence of BrdU using FlowJo software. Plots depict BrdU incorporation versus DAPI in both populations. Population of ES cells in G1, S, and G2 phase are also depicted. (**c**) Analysis of Z-stack images of RPA protein. Z-stack images were recorded with an interval of 0.2 μm between each plane (20–30 planes total), and then processed to a 3D projection by NIS-AR software (Nikon). 3D-reconstruction images showing RPA foci under each indicated condition with immunostaining for RPA. (**d**) Accumulation of larger ssDNA gaps in the RAD51 knockdown cells. The diameters of RPA foci were quantified in normal cells and the siRAD51 treated-cells from (**c**). About 200 cells were counted for each experiment. Error bars indicate the mean ± SD. ***P < 0.001 (Student’s t-test). (**e**) Immunoblot analysis of Rad51, TopBP1, PCNA, PLK1, and Chk2 proteins. Cells were collected for western blot analysis 48 h after siRNA transfection, and protein levels were detected with the corresponding antibodies. (**f**) Immunoblot analysis of cell cycle proteins CENP-F and CyclinA1 in siCtrl and siRAD51 cells.

We used RNA interference to inhibit Rad51 protein expression in mES cells and investigate its role in cell cycle progression (Fig. 4). The expression level of Rad51 protein was reduced by 86.7% in mES cells transfected with small interfering RNA (siRNA) against Rad51 (siRAD51) compared to expression in mES cells transfected with control siRNA (siCtrl) (Fig. 4a). To determine whether Rad51 protein expression is involved in DNA replication, we performed a BrdU-incorporation assay after transfecting mES cells with siRad51 (Fig. 4b). *RAD51* knockdown in mES cells caused cell cycle arrest at the G2/M phase, leading to reduced cell proliferation (Fig. 4b). The percentage of cells in G2/M phase increased by approximately 1.58-fold in siRAD51-transfected mES cells when compared with this percentage in siCtrl-transfected mES cells as determined by BrdU positivity (Fig. 4b). However, quantification of the BrdU index revealed that the population of cells in S phase did not change significantly following siRAD51 transfection when compared with the S-phase population of siCtrl-treated mES cells (Fig. 4b). We also monitored the size of ssDNA directly by staining chromosomes with anti-RPA. In siRNA control cells, the foci diameter exhibited a 0.27 ± 0.17 µm. When *RAD51* gene expression was suppressed in ES cells, the foci diameter increased to 0.39 ± 0.23 µm, indicating appearance of larger unrepaired ssDNA gaps upon Rad51 depletion (Fig. 4c,d). Having confirmed that Rad51-dependent HR likely acts in post-replication stages to repair DNA breaks with ssDNA, we next examined whether siRAD51 affected the expression of several proteins required for DNA replication and cell cycle progression, including TopBP1, PCNA, Plk1, and Chk2 (Fig. 4e). The expression levels of these factors were unchanged in siRad51-transfected mES cells compared to their levels in siCtrl-transfected mES cells. Therefore, the protein level of Rad51 in mES cells did not influence the expression of these factors, which are essential for formation of the pre-initiation complex and for replication-related activities (Supplementary Fig. S3). However, we previously observed that γH2AX and Chk1 phosphorylation occurred in Rad51-knockdown ES cells^3^, and observed here that CENP-F and Cyclin A1, the cell cycle marker proteins for G2/M, were increased in Rad51-knockdown ES cells (Fig. 4f), suggesting that checkpoint signals could be activated in response to DNA breaks at the G2/M phase transition. Thus, Rad51 may play a critical role in protecting replication forks at the end of DNA replication, specifically during the transition from late S phase to G2, to prevent DNA break formation or replication folk collapse.

To determine whether Rad51-dependent machinery was involved in ES cell-specific cellular progression, we examined the role of Rad54, a key accessory factor of Rad51, in the G2/M transition. First, we expressed a short hairpin RNA (shRNA) against *RAD54* in a mES cell line carrying a doxycycline-inducible shRAD54 plasmid and observed cell cycle progression. This system enabled the inhibition of Rad54 expression in the presence of doxycycline (Supplementary Fig. S4). The induction of shRAD54 arrested ES cells at the G2/M phase, leading to reduced proliferation as shown upon *RAD51* knockdown (Supplementary Fig. S4). Consistent with *RAD51* knockdown data, Rad54 acted critically to mediate the HR pathway (or ssDNA gap repair) throughout late S-to-G2/M phase during the end of DNA replication. Thus, depleting Rad51 or Rad54 likely induces G2/M arrest because of the presence of unrepaired DNA breaks after the completion of DNA replication.

### Serum starvation reduces levels of HR factors, but not PCNA, and stimulates the G2/M checkpoint

**Figure 5:**
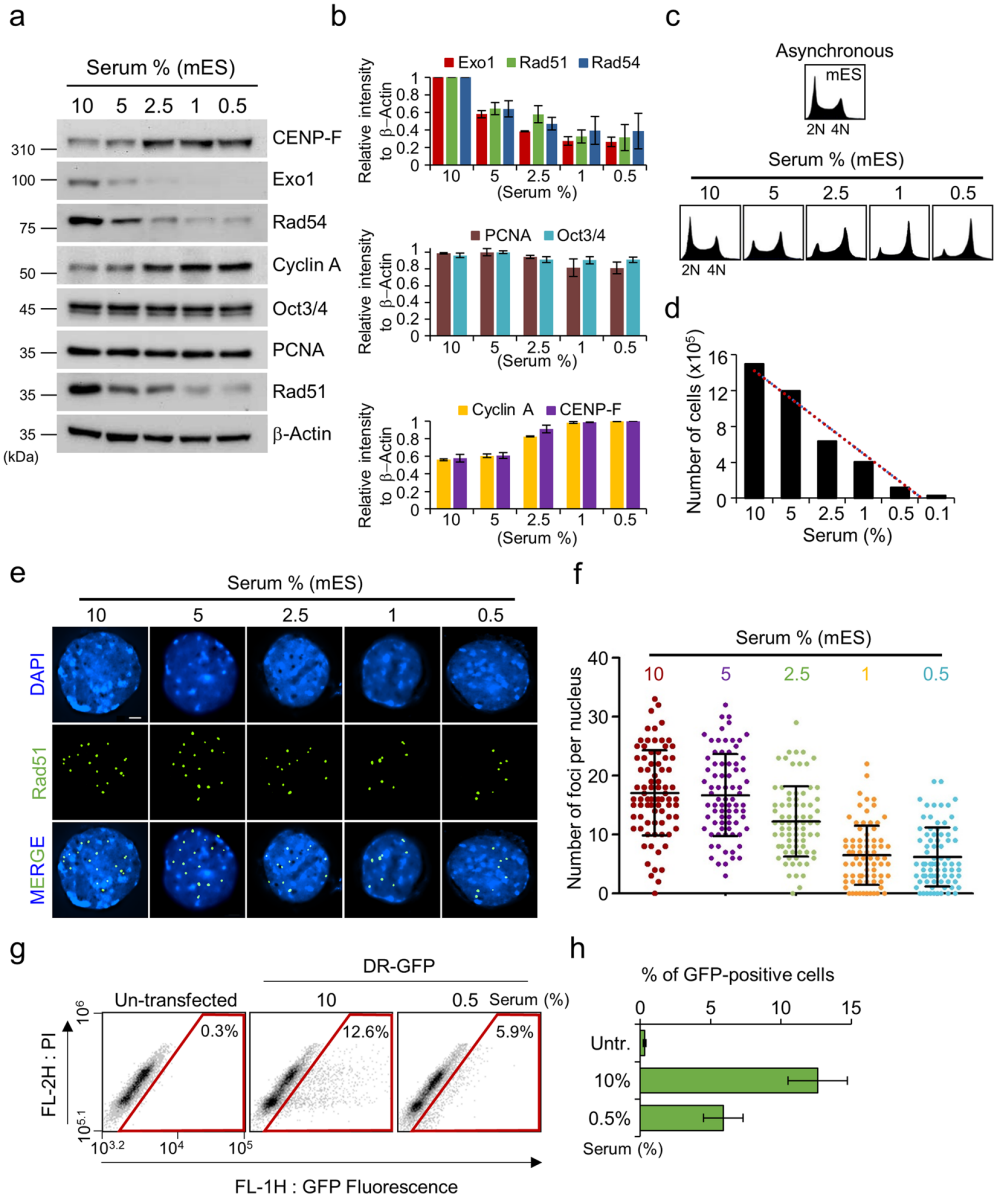
Effect of the culture medium serum concentration on cell cycle progression and expression of HR factors in mES cells. (**a**) Immunoblot analysis of Exo1, Rad54, Rad51, PCNA, CENP-F, Cyclin A1, and Oct3/4 in mES cells. The expression of HR factors, CENP-F, and Cyclin A1 in mES cells was dependent on the serum concentration in the culture medium. β-actin was used as a loading control. (**b**) Quantification of Exo1, Rad54, Rad51, PCNA, CENP-F, Cyclin A1, and Oct3/4. The protein levels in (**a**) were quantified relative to the intensity of the β-actin signals. Error bars indicate the mean ± SD. (**c**) FACS analysis of mES cells in various serum concentration. Exponentially growing mES cells were cultured in medium with serial dilutions of serum at the indicated concentrations, and the cell cycle pattern was analyzed by FACS. mES cells grown with the indicated serum concentrations were harvested at 48 h, and the cell cycle patterns were profiled using a FACSCalibur flow cytometer. (**d**) Quantification of mES cell numbers in various serum concentrations (0.1, 0.5, 1, 2.5, 5, and 10% serum concentration in culture medium). (**e**) Representative images showing Rad51 foci in mES cells grown in the presence of different serum concentrations. mES cells were immunostained with antibodies against Rad51. (**f**) Quantification of Rad51 foci per nuclei after culture in the indicated serum concentration. More than 80 nuclei were counted for each experiment. Error bars indicate the mean ± SD. (**g**) Analysis of pDR-GFP HR assay. mES cells carrying the GFP-HR plasmid were transfected with I-SceI plasmid on the culture medium with the indicated serum concentration (untreated, 10%, and 0.5% serum). (**h**) The proportions of GFP-positive mES cells in (**g**) were quantified. Error bars indicate the mean ± SD.

Proliferating cell nuclear antigen (PCNA) functions as a processivity factor that holds DNA polymerases delta and epsilon on the DNA template during replication^3, 27^. PCNA as a proliferative marker is highly expressed throughout the cell cycle, and most abundantly during DNA replication. In addition, PCNA levels have are serum concentration-dependent^28^. Previous studies have shown that a low serum concentration results in reduced cell division rate or induces a reversible cell cycle synchronization^29^. Thus, ES cells likely express PCNA abundantly during cell proliferation, along with consistent expression of HR factors, and may utilize regulatory mechanisms involving HR and DNA replication against stress caused by nutrient deficiency, thereby promoting DNA repair and cell cycle progression. To investigate the possibility that cell cycle progression is tightly coupled with cellular signaling that directs the expression of HR factors and PCNA, we analyzed the levels of HR factors and PCNA in mES cells under serum starvation by immunoblot analysis (Fig. 5a). Reducing the serum concentration in the culture medium inhibited the expression of Rad51, Rad54, and Exo1, with Exo1 expression being the most sensitive to changes in serum concentration (Fig. 5a,b). However, mES cells expressed Oct3/4, a stemness marker, at the similar levels regardless of serum level (Fig. 5a,b). These results confirmed that low serum concentration did not affect maintenance of stemness in mES cells, although the expression of HR factors was suppressed. In addition, PCNA expression remained high compared to the expression of Rad51, Rad54, and Exo1, even in the presence of just 0.5% serum (Fig. 5a,b). Accordingly, the low levels of HR proteins produced by mES cells during serum starvation probably arrest the cell cycle a G2/M phase (Fig. 5c). Thus, constitutive expression of HR factors might help bypass the G2/M checkpoint and maintain high levels of cells in S phase. To test this hypothesis, we reduced the serum concentration in the growth medium of mES cells to 0.5% and analyzed the cell cycle phases by FACS analysis (Fig. 5c). As expected, the cell cycle profile of mES cells grown with a low serum concentration exhibited G2/M arrest in a similar manner as mES cells treated with siRAD51 (Fig. 5c). However, no significant changes were seen in cell cycle profile of MEFs (Supplementary Fig. 5). In addition, although mES cells were gradually introduced to the reduced serum concentration over a period of 2 days, the cell growth rate appeared to be delayed and levels of G2/M marker proteins, CENP-F and Cyclin A1, were markedly increased compared to the levels exhibited in 10% serum (Fig. 5a-c). However, no growth was observed at 0.1% serum concentration (Fig. 5d). Collectively, these data indicated that the cell cycle profile of mES cells was dependent on serum concentration and was likely changed by reducing levels of HR factors. We also examined Rad51 focus formation under reduced-serum conditions (Fig. 5e,f). Rad51 proteins still assembled at chromosome break sites in cells grown in the presence of 0.5–5% serum, but the total number of foci decreased in proportion to the serum concentration (Fig. 5f). To access HR activity in mES cells on the reduction of serum concentration, we used a more direct method using green fluorescence protein (GFP)-HR assay system containing I-SceI expression gene. In mES cells at 10% serum concentration, ~12.6% GFP-positive cells were observed. However, in 0.5% serum concentration, the number of mES cells with GFP-positive signals was reduced to ~5.9% (Fig. 5g,h).

### Caffeine treatment promotes the loss of previously assembled HR protein foci involved in strand exchange and increases DNA damage sensitivity

**Figure 6:**
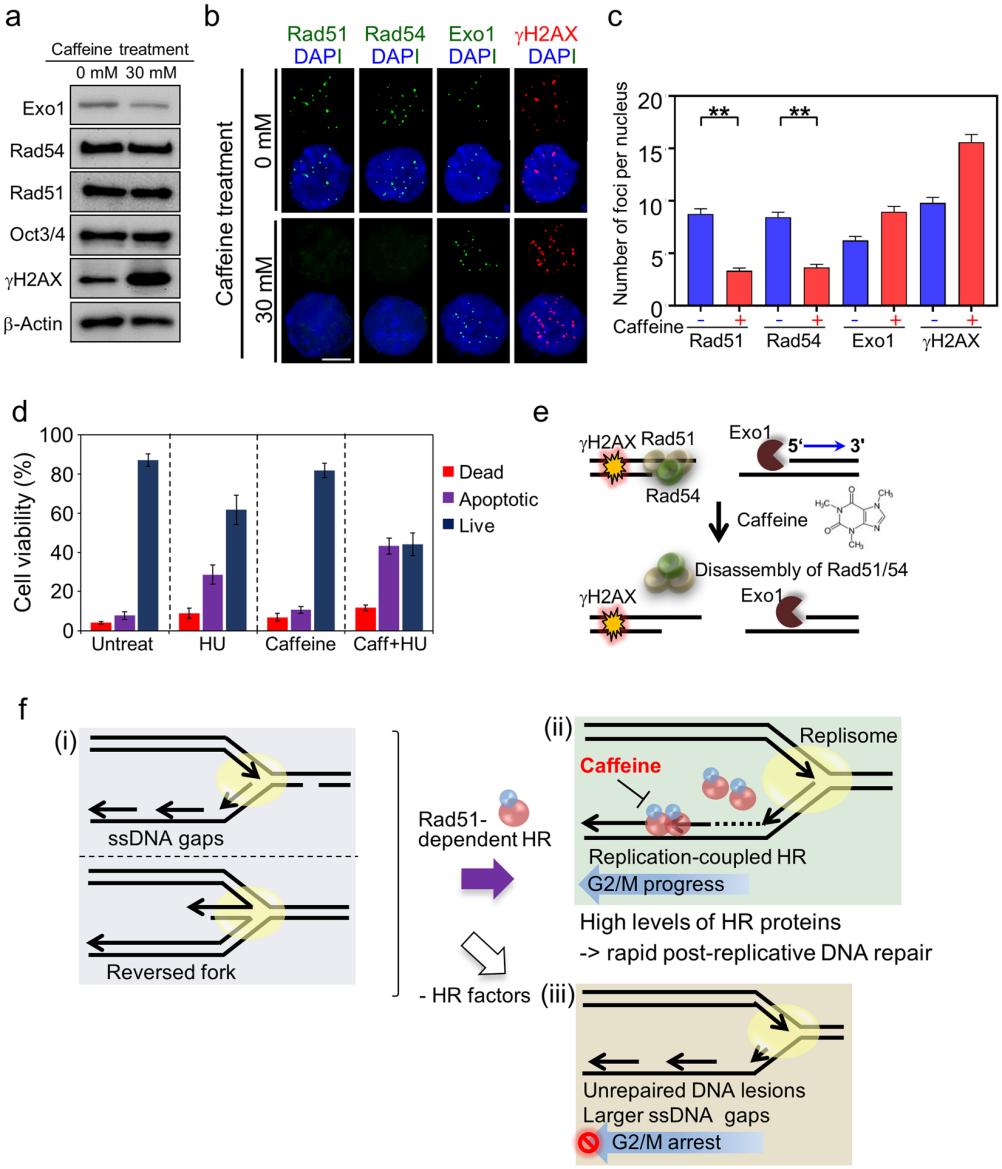
Caffeine treatment promotes the disassembly of Rad51 and Rad54, but not Exo1 and γH2AX, on DNA breaks in mES cells. (**a**) The levels of Exo1, Rad54, Rad51, Oct3/4, and γH2AX in mES cells were detected in the presence or absence of 30 mM caffeine by western blot analysis. mES cells exhibited similar levels of HR factor expression in the presence or absence of caffeine. β-actin was used to show relative loading of cell lysates. (**b**) Representative images showing the average numbers of Rad51, Rad54, Exo1, and γH2AX foci formed in the presence or absence of caffeine. mES cells were treated with 30 mM caffeine for 4 h, fixed, and then immunostained with anti-Rad51, anti-Rad54, anti-Exo1, and anti-γH2AX antibodies. (**c**) Quantification of Rad51, Rad54, Exo1, and γH2AX foci. Caffeine inhibited Rad51-mediated strand exchange through interference with Rad51-ssDNA nucleofilament formation, thereby suppressing HR. In contrast, focus formation by Exo1 and phosphorylated γH2AX was not affected by caffeine treatment. Error bars indicate the mean ± SEM. ***P ≤ 0.001 (Student’s t-test). (**d**) Analysis of cell viability in response to caffeine and/or HU treatment. The percentages of live, apoptotic, and dead cells were measured under DNA-damaging conditions with the indicated chemical treatments. (**e**) Cartoon for caffeine-induced disassembly of Rad51 and Rad54 proteins. In the presence of caffeine, Exo1 and γH2AX are highly accumulated on DNA break sites, but Rad51 and Rad54 are dissociated from the DNA breaks. (**f**) Proposed model for HR-mediated maintenance of genome integrity in mES cells. (i) Accumulation of ssDNA gaps at the S-to-G2/M transition or reversed forks in mES cells. (ii) HR factors are constitutively expressed throughout the cell cycle. Therefore, the HR-mediated repair pathway can respond immediately to the accumulation of ssDNA gaps and DNA damages to promote efficient replication. Caffeine inhibits the assembly of Rad51 and its accessory factors on ssDNA, which could cause a defect in the Rad51-dependent HR pathway that promotes normal proliferation and self-renewal. (iii) The absence of Rad51 might lead to unrepaired DNA lesions or the accumulation of larger ssDNA gaps, which induces arrest at the G2/M phase.

Biochemical analyses have shown that caffeine inhibits the ssDNA-binding activity of Rad51, which could also indicate that caffeine directly inhibits HR^30^. To further investigate whether caffeine abolished focus formation of HR factors in ES cells, the assembly of Rad51, Rad54, Exo1, and γH2AX foci on chromosome break sites was examined by immunofluorescence in mES cells exposed to caffeine (Fig. 6). Immunoblot analysis revealed no significant changes in expression levels of Rad51, Rad54, and Exo1 upon caffeine treatment (Fig. 6a). When ES cells were treated with 30 mM caffeine for 4 h, the number of Rad51 foci per nucleus decreased from 8.8 ± 0.50 to 3.2 ± 0.26, and the number of Rad54 foci per nucleus decreased from 8.4 ± 0.49 to 3.6 ± 0.31 (Fig. 6b,c). These findings suggested that Rad51 and Rad54 dissociated from ssDNA upon caffeine exposure. In contrast, the average number of Exo1 foci per nucleus increased following caffeine treatment from 6.2 ± 0.40 to 8.9 ± 0.52 because the number of γH2AX foci (unrepaired DNA damage foci) per nucleus increased from 9.8 ± 0.53 to 15.6 ± 0.73, consistent with the observed decrease in DSB repair (reduced Rad51 and Rad54 foci) (Fig. 6c). Thus, caffeine inhibited the formation of Rad51 and Rad54 foci, but not the formation of γH2AX and Exo1 foci on ssDNAs, which led to incomplete HR. Further, in the presence of caffeine and the DNA-damaging agent HU (4 mM; Supplementary Fig. S6), the percentage of apoptotic cells increased from 28.6% to 43.2%, and the percentage of dead cells increased from 9.2% to 11.8% (Fig. 6d and Supplementary Fig. S7). Thus, we conclude that caffeine suppressed HR by inhibiting Rad51-mediated strand exchange and might affect the HR-mediated repair pathway, which is crucial for maintaining genome stability in ES cells (Fig. 6e). Previous results showed that caffeine treatment affected HR independently of its inhibition of checkpoint kinases, which are essential for the HR pathway^30, 31^. Accordingly, the formation of both Rad51 and Rad54 foci were significantly decreased following caffeine treatment, which is expected to impair HR in ES cells. In contrast, the γH2AX and Exo1 foci numbers slightly increased, as the population of cells in S phase increased (Fig. 6c,e; Supplementary Fig. S2) or additional DNA breaks could be generated following caffeine treatment, and these might result in incomplete HR and increased sensitivity against chromosomal instability (Supplementary Fig. S7). Thus, caffeine could be used to control cellular progression in ES cells by inhibiting the formation of Rad51-ssDNA nucleofilaments, but not DSB resection.

## Discussion

In this study, we provided evidence that ES cells controlled the expression of HR factors through specific mechanisms that differ from those utilized in differentiated cells. Furthermore, the expression levels of HR factors in ES cells did not change significantly throughout the cell cycle or as a result of DNA breaks induced by exogenous DNA-damaging agents. Interestingly, caffeine treatment in ES cells resulted in Rad51/Rad54 dissociation from ssDNA templates on chromatin, thereby inhibiting Rad51-catalyzed strand exchange.

Exposure of cells to DNA-damaging conditions either directly or indirectly results in global DNA breaks and stalled replication. Although DNA-damaging conditions produce similar levels of DNA breaks in differentiated cells and ES cells, ES cells constitutively express multiple HR proteins, allowing these cells to mediate DNA repair in the S and G2 phases of the cell cycle. In contrast, the levels of HR proteins were apparently reduced during ES cell differentiation (Kim and Choi, unpublished). It has also been reported that the G1/S DNA-damage checkpoint in ES cells may be activated upon DNA damage and prevent entry into S phase^25^. Our data also demonstrated that various DNA-damage reagents might stop cells from entering the S phase to prevent normal DNA replication or induce cell cycle arrest in the late S or G2 phases. Although the levels of HR proteins were unchanged upon treatment with DNA damage-inducing chemicals, the number of HR foci increased, suggesting that the existing HR proteins were rapidly activated for HR-mediated DNA-break repair (Fig. 6f). One possible explanation is that HR proteins are highly expressed, but remain inactive until post-translational modifications are triggered by a DNA-damage signal.

Under conditions in which spontaneous DNA breaks occur, proliferative cells can overcome the replication stress by several DNA repair mechanisms, including NHEJ, microhomology-mediated end joining (MMEJ), and HR. Post-replication repair (PRR) is the repair of DNA damage that occurs after DNA replication. Some representative genes include HR factors: Rad51, Rad52, Rad54, BRCA1, BRCA2, BLM, and MRN complex (Mre11, Rad50S, and Nbs1 in human). ES cells exhibit a prolonged S phase and rapid cell cycle. Further, HR-related proteins are abundantly expressed throughout the cell cycle, implying that HR is probably essential to maintain ES cell-specific cell cycle behavior. As shown in Fig. 6f, depletion of RAD51 in mES cells resulted in cell cycle arrest at the G2/M phase and accumulation of unrepaired larger ssDNA, suggesting that the Rad51-dependent pathway could be relevant to the later stages of DNA replication in ES cells. Further, caffeine treatment suppressed HR by interfering with Rad51-mediated recombination, resulting in defective DNA damage repair in ES cells. Thus, DNA breaks caused by replication machinery might result in G2/M checkpoint stalling and should be repaired properly by HR to avoid stem cell tumorigenicity and cell death. Notably, HR-mediated PRR is the most effective pathway that allows damaged DNA to be repaired using a sister template (after DNA replication) for the self-renewal of ES cells.

PCNA functions together with Ki-67^32^ as a loading platform for various DNA replication factors in actively replicating cells^33, 34^. Remarkably, PCNA and HR factors were expressed at a constant level throughout the cell cycle in this study, indicating that actively proliferating ES cells constitutively expressed components of DNA repair and DNA replication machinery. In response to reduced serum concentration, the expression levels of HR factors decreased with decreasing serum concentration, and cell cycle progression of ES cells was arrested at the G2/M checkpoint. Thus, Rad51-dependent HR is essentially related to the proliferation rate of ES cells. However, unlike the HR factors, whose levels decreased during serum starvation, PCNA expression did not decrease under reduced serum concentrations. In addition, besides the roles of PCNA in cell cycle control, it is also involved in post-replication repair and some DNA-repair pathways including base-excision repair, nucleotide-excision repair, and mismatch repair^35–37^. In agreement with this result, ES cells may utilize multiple mechanisms to control the activation of the constitutive expression of key factors, which are involved in cell cycle-regulatory processes and DNA-repair pathways. Thus, it could be interesting to further investigate how serum-dependent nutrient conditions regulate the expression of HR factors and ES cell-specific replication machinery.

ES cells offer an unprecedented opportunity to better understand cellular differentiation and to establish preclinical models for the study of human diseases. Our findings suggested that ES cells effectively regulate essential factors of the HR machinery to repair DNA breaks and maintain genomic integrity, regardless of the cell cycle phase. Key findings of the present study support the hypothesis that the abundance of HR factors expressed in ES cells facilitates the immediate HR-mediated, PRR of ssDNA gaps, fork reversals, and DNA damage through a mechanism that does not affect cell growth rate. Alternatively, Rad51, Rad52, and Rad54 might cooperatively inhibit continued DNA-break resection that would yield unrepaired ssDNA gaps in ES cells^38, 39^. These mechanisms may also help preserve genomic integrity and efficient cell cycle progression by utilizing high-fidelity HR to repair intrinsic DNA breaks and overcome replication stress-induced arrest of proliferation in ES cells. Therefore, these findings could provide a centralized resource to develop optimal strategies for ES cell-based cellular therapies.

## Methods

### Cell culture and synchronization

The J1 murine ES cells were prepared as described previously^3^. For S-phase synchronization, cells were treated with 2 mM thymidine for 16 h, and then washed with 2 mL phosphate-buffered saline (PBS). For cell cycle release, fresh medium was added to cells for 6 h, and then another 2 mM thymidine treatment was performed before the cells were incubated again for 16 h. Cells were then washed with PBS and fresh medium was added. Cells were harvested 2.5 h after release from the thymidine block.

### BrdU FACS analysis

Cells were pulsed with 5-bromo-2′-deoxyuridine (BrdU) at a final concentration of 10 μM for 20 min to ensure sufficient BrdU incorporation. The cells were harvested and washed briefly with PBS, and then fixed with 70% ethanol for 2 h. The fixed samples were denatured with denaturation buffer (2 M HCl and 0.5% Triton X-100 in PBS) for 30 min. After washing with PBS, the samples were recovered in neutralization buffer (0.1 M Na_2_B_4_O_7_-H_10_O_2_, pH 8.5) for another 30 min and incubated with an anti-BrdU antibody (Cat. No. ab6326; Abcam), which was diluted in 1% BSA and 0.5% Tween 20 in PBS, for 1 h at 25 °C. Next, the samples were incubated for 1 h with a FITC-conjugated secondary antibody. After washing with PBS, the samples were stained for 1 h in propidium iodide with RNase A (Sigma). Finally, the samples were analyzed using a FACSCalibur flow cytometer (Becton Dickinson) and quantified with FlowJo software (Tree Star, Inc.).

### Induction of DNA damage

To examine protein expression and intracellular localization of HR factors, as well as the cell cycle profiles in response to various types of DNA damage, mES cells were treated with the following: 4 mM hydroxyurea (HU) or 5 μg/mL aphidicolin (Aph) for 16 h; 200 nM camptothecin (CPT), 10 μM etoposide (Eto), 10 μM cisplatin (Cis), or 30 mM caffeine for 4 h.

### Cell death analysis

To analyze cell death, J1 cells were briefly washed with cold PBS. Harvested cells were suspended in PBS containing 84 nmol/L thiazole orange (TO; BD) and 4.3 μmol/L PI (BD), and were incubated for 5 minutes at room temperature. Cells were analyzed using a FACScalibur flow cytometer (Becton Dickinson) and quantified with FlowJo 10.2 v software (FlowJo, LLC).

### Serum starvation

To slow the proliferation rate of mES cells, the concentration of horse serum in the culture medium of J1 cells was serially reduced from 10% to 1%, 0.5%, and finally 0.1%.

### Western blot analysis

Samples for western blot assays were prepared as described previously^3^. The following primary antibodies were used: Rad51 (diluted 1:2,000; Cat. No. sc-8349; SCBT); Rad54 (diluted 1:2,000; Cat. No. sc-374598; SCBT); PCNA (diluted 1:2,000; Cat. No. sc-56; SCBT); Plk1 (diluted 1:3,000; Cat. No. sc-17783; SCBT); Oct3/4 (diluted 1:3,000; Cat. No. sc-5279; SCBT); Chk2 (diluted 1:3,000; Cat. No. sc-9064; SCBT); α-Tubulin (diluted 1:5,000; Cat. No. sc-8035; SCBT); β−Actin (diluted 1:10,000; Cat. No. ab8226; Abcam); Cyclin A (diluted 1:2,000; Cat. No. sc751; SCBT); CENP-F (diluted 1:1000; Cat. No. ab5; Abcam); γH2AX (diluted 1:2,000; Cat. No. 2577; CST); TopBP1 (diluted 1:3,000; Cat. No. sc-271043; SCBT); and Exo1 (diluted 1:1,000; Cat. No. MS-1534; Thermo Fisher). Immunoblot signals were developed with a WEST-ZOL detection system (Cat. No. 16024; iNtRON Biotechnology). The relative amount of each protein was quantified using ImageJ software.

### Immunofluorescence analysis

For immunofluorescence, ES cells were fixed with 4% paraformaldehyde and permeabilized with 0.2% Triton X-100 (Sigma). Antibodies against the following proteins were used: Rad51 (diluted 1:200; Cat. No. sc-8349; SCBT), Rad54 (diluted 1:200; Cat. No. sc-374598; SCBT), Exo1 (diluted 1:100; Cat. No. MS-1534; Thermo Fisher), and γH2AX (diluted 1:200; Cat. No. 2577; CST). The cells were washed twice with PBST for 5 min and incubated with Cy3 anti-Rabbit (diluted 1:500; Cat. No. 111-165-003; Jackson) and Alexa Fluor 488 anti-Mouse (diluted 1:500; Cat. No. 111-545-003; Jackson) as secondary antibodies. Images were acquired with an Eclipse Ti-E fluorescence microscope (Nikon, Japan) with a DAPI filter and fluorescent channels.

### RNA interference

All small interfering RNA (siRNA) used were part of a commercially available siGENOME SMART pool (Dharmacon). Four individual siRNAs targeting 4 oligonucleotides with following sequences were mixed: 5′-CAUCAUCGCUCAUGCGUCA-3′, 5′-UGUCAUACGUUGGCUGUUA-3′, 5′-GGUAAUCACCAACCAGGUA-3′, and 5′-GAGAUCAUACAGAUAACUA-3′.

Cells were treated with a final siRNA concentration of 200 nM in serum-free media using DharmaFECT (Dharmacon) transfection reagents to deliver siRNA into ES cells. Cells were incubated at 37 °C and 5% CO2 for 48 h post-transfection.

### RNA isolation

Total RNA from cultured cells was extracted using an RNeasy Mini Kit (Qiagen), according to the manufacturer’s protocol. Total RNA was quantified using a NanoDrop 2000 Spectrophotometer (Thermo Fisher, DE, USA).

### Data analysis

To determine the expression level of gene regions and relationships between a gene product and its biological process, we used the fragments per kilobase per million (FPKM) method, utilizing the Gene Set Enrichment Analysis (GSEA) with GSEA java software. Transcript analysis was performed using StringTie (https://ccb.jhu.edu/software/stringtie/).

## Electronic supplementary material

Supplementary information
