# Supplementary material for: The Homologous Recombination Machinery Orchestrates Post-replication DNA Repair During Self-renewal of Mouse Embryonic Stem Cells
Source: Sci Rep. 2017 Sep 14;7:11610. doi: 10.1038/s41598-017-11951-1 (PMC5599617; doi:10.1038/s41598-017-11951-1)

The Homologous Recombination Machinery Orchestrates Post-replication DNA Repair During Self-renewal of Mouse Embryonic Stem Cells

Eui-Hwan Choi[1], Seobin Yoon[1], Kyung-Soon Park[2], and Keun P. Kim[1*]

[1] Department of Life Sciences, Chung-Ang University, Seoul 156-756, Korea
[2] Department of Biomedical Science, CHA University, Seoul, Korea

Supplementary figure legends

Supplementary Figure 1. RNA-Seq analysis for asynchronous and S-phase synchronous cultures. (**A**) Heat map of the fold-change values in gene expression of HR factors in synchronized at S phase. The colored bar is a log_2-scale representation of the fold-changes of transcript levels observed between asynchronous and synchronous cultures. (**B**) Heat map of the transcript-level fold-changes for genes related to non-homologous end joining (NHEJ) in cells synchronized in S phase. The colored bar is a log_2-scale representation of the fold-changes of transcript levels observed between asynchronous and synchronous cultures. (**C**) Fold-change values of transcripts for genes related to NHEJ in cells synchronized in S phase. The values of the dotted lines (± 0.32 log_2) represent the fold-change in β-actin expression from four-independent RNA-Seq experiments. (**D**) Analysis of qPCR for HR genes in ES cells synchronized in S-phase. Three-independent experiments of each sample were run and the Ct values averaged. Error bars indicate the mean ± SEM. (**E and F**) Fold-changes in transcript levels of synchronized ES cells in S-phase. Changes in HR gene expression analyzed by RNA-Seq compared against qPCR results. Log_2 values of fold-changes measured in terms of the FPKM compared with fold-changes in mRNA expression detected by qPCR analysis.

Supplementary Figure 2. FACS analysis of cell viability in response to DNA damage-inducing agents. (**A**) The cell cycle distribution of ES cells in the presence of chemical reagents. (**B**, **C**) Analysis of cell viability in response to DNA damage-inducing agents. The percentages of live, injured, and dead cells were measured after exposure to various chemical treatments.

Supplementary Figure 3. Changes in expression levels of regulatory genes in response to *RAD51* knockdown. (**A**) The fold changes of transcript levels of genes involved in pre-replication, pre-initiation, and replication initiation. The color bar shows a log_2-scale representation of the fold-changes of transcript levels in response to *RAD51* knockdown. (**B**) Significantly differentially expressed gene classes involved in apoptosis, the cell cycle, DNA replication, the G1/S transition, and cell proliferation.

Supplementary Figure 4. Analysis of cell cycle and protein expression in mES cells after the induction of shRAD54 expression

(**A**) Rad54 expression in mES cells were inhibited by induction of an shRNA against

RAD54 via a doxycycline-inducible pSingle-tTs plasmid. (**B**) FACS analysis of cell cycle profiles in the presence or absence of doxycycline. Doxycycline (final concentration, 1 μg/ml) was added to the culture media to induce shRAD54 expression.

Supplementary Figure 5. Analysis of MEF cell cycle on the reduction of serum concentration. FACS analysis of MEFs in various serum concentration. MEF cells grown with the indicated serum concentrations were harvested at 48 h, and the cell cycle patterns were profiled using a FACSCalibur flow cytometer.

Supplementary Figure 6. Analysis of cell cycle and cell viability in response to HU-reagent. (**A**) FACS analysis of cell cycle on diverse concentration of HU reagent. mES cells were treated with HU (untreated, 0.2 mM, 0.5mM, 1 mM, 2 mM, 4 mM, 6 mM and 8 mM) as described in Materials and Methods and stained with PI. (**B**) FACS analysis of cell viability in the presence or absence of HU reagent. mES cells were stained with TO and PI for quantification of percent of live, and dead cells. (**C**) Cell numbers on various HU concentration. mES cells were treated with HU and the viable cells were counted by FACS.

Supplementary Figure 7. FACS analysis of cell viability in response to caffeine in the presence of absence of HU-reagent. (**A**) The percentages of live, apoptotic, and dead cells were determined under DNA-damaging conditions with the indicated chemical treatments (4 mM HU and 30 mM caffeine). (**B**) Essential roles of Rad51-dependent HR relevant to cell cycle progression and evasion of programmed cell death.

Supplementary Figure 8. (**A, B**) Full-length blots of Exo1, Rad54, Rad51, PCNA, and α-tubulin in mES and MEF (shown in Fig. 1C). The full-membrane blots cropped and incubated with the indicated antibodies. (**C, D**) Less expose blots of Exo1, Rad51, Rad54, and α-tubulin shown at the bottom. The presence of another band besides the predicted one after the incubation with a specific antibody is due to previous antibody incubations

Supplementary Figure 9. (**A, B**) Full-length blots of HR factors, Exo1, and α-tubulin under DNA-damaging conditions with the indicated various chemical treatments (shown in Fig. 3C). (**C**) Short expose blot of HR factors in mES cells.

Supplementary Figure 10. (**A-C**) Full-length blots of genes related to DNA repair,

replication, or cell cycle under knock-down of Rad51 in mES cells (shown in Fig. 4A, E and F). The full-membrane blots cropped and incubated with the indicated antibodies.

Supplementary Figure 11. Full-length blots of genes related to DNA repair, replication, cell cycle, and stemness marker under serum starvation in mES cells (shown in Fig. 5A). The presence of another band besides the predicted one after the incubation with a specific antibody is due to previous antibody incubations. The full-membrane blots cropped and incubated with the indicated antibodies.

Supplementary Figure 12. Full-length blots of genes related to DNA repair, replication, stemness and DNA damage marker under cell stress conditions with the indicated caffeine treatment. (shown in Fig. 6A).

Supplementary tables

Supplementary Table S1. Primer sequences used in quantitative polymerase chain reaction

Gene	Sequence
MRE11	Forward: GCC CCC TTT TCC TGA GAG TT
	Reverse: GTG GAT CTG TGG GGC TCA TT
RAD50	Forward: CCC CTG GCA CTG AGT AAA GG
	Reverse: AAT GAT GGT CGT CTT CCC `CG
DNA2	Forward: GCC AGA TGC TGA TCG GTA CA
	Reverse: CCA TCG CTT GGC AGA GAG AG
EXO1	Forward: TAA ACA CGT CGA GCC TGT CC
	Reverse: CAG AGC CCA GGA ACC TTG TT
BLM	Forward: CCC AGA CCG GAG TAG AGA CC
	Reverse: CCA TGA TCC TCA TCT GGC ATC C
RPA1	Forward: CTG AGG AAG TTA AGC CAG AAA GT
	Reverse: CAA ATG GCT TTG AGG CAC CA
RPA2	Forward: GAA ATC GAG AGT CCG AGC CC
	Reverse: GAG GTG ACC AGC GAC TTT CA
RPA3	Forward: GAT GGA GCC ACT TGA CGA GG
	Reverse: CTT CAG TGT AGC TTC CTG GCA
RAD52	Forward: TCA GGG GGC TAA GGA CAT CA
	Reverse: AAG TAC TGC CGC ATG CTT GG
RAD51	Forward: GCA ACT GAG TTT CAC CAG CG
	Reverse: ATG GGG AGC TGG CAT GTA AC
RAD51AP1	Forward: CAA AGA GAA AAG CAC TGA CAA ACA
	Reverse: TCT GTG ATC TTG TCC AAA TCT TCT
RAD54l	Forward: GAT GTC GGT CCA GCT CTG CAT

	Reverse: TTA GGA GTT ACT GTC CCA GGC T
BRCA1	Forward: AGG CTT GAC CCC CAA AGA AG
	Reverse: CCG GAC CAC CCA TGA ATA GC
BRCA2	Forward: CTA ACG TAC TTC CTT ACC GAG CA
	Reverse: CTG TGC TGC ATC TCG CCT TA
PCNA	Forward: TGC TCT GAG GTA CCT GAA CT
	Reverse: TGC TTC CTC ATC TTC AAT CT
POLn	Forward: AGC TTA TAT GAA AAA GGG CTC CA
	Reverse: CAA AAC AGT GAC TCC AAG GCA AG
OCT3/4	Forward: TCT TTC CAC CAG GCC CCC G
	Reverse: GGC GGA CAT GGG GAG ATC C
GAPDH	Forward: AAG GTC ATC CCA GAG CTG AA
	Reverse: CTG CTT CAC CAC CTT CTT GA

Supplementary Table S2. **Gene-expression levels of ES cells in synchronized culture at S phase.**

Gene symbol	Ensembl ID	Fold-change	Log2 fold-change	locus
MRE11	ENSMUSG00000031928	1.131	0.177	chr9:14784706-14834417
RAD50	ENSMUSG00000020380	1.060	0.084	chr11:53649518-53707319
DNA2	ENSMUSG00000036875	0.835	-0.261	chr10:62947028-62974188
EXO1	ENSMUSG00000039748	0.824	-0.279	chr1:175880777-175911396
NBN	ENSMUSG00000028224	0.745	-0.425	chr4:15957966-15992589
TOP3A	ENSMUSG00000002814	0.965	-0.051	chr11:60740058-60777365
BLM	ENSMUSG00000030528	0.821	-0.284	chr7:80454992-80535119
RPA1	ENSMUSG00000000751	0.970	-0.045	chr11:75300258-75348383
RPA2	ENSMUSG00000028884	1.015	0.022	chr4:132768359-132778746
RPA3	ENSMUSG00000012483	1.056	0.079	chr6:8255935-8259141
RAD52	ENSMUSG00000030166	0.942	-0.086	chr6:119902697-119922823
RAD51	ENSMUSG00000027323	1.144	0.195	chr2:119112816-119136070
RAD51AP1	ENSMUSG00000030346	1.144	0.194	chr6:126923418-126939555
RAD54l	ENSMUSG00000028702	0.990	-0.014	chr4:116075464-116123690
BRCA1	ENSMUSG00000017146	1.072	0.100	chr11:101488763-101551955
BRCA2	ENSMUSG00000041147	0.799	-0.324	chr5:150522622-150569749
PCNA	ENSMUSG00000027342	1.090	0.124	chr2:132249285-132253180
REV3l	ENSMUSG00000019841	0.964	-0.053	chr10:39732159-39875205
POLn	ENSMUSG00000045102	0.865	-0.210	chr5:34007199-34169448
TOPBP1	ENSMUSG00000032555	1.013	0.018	chr9:103305326-103350427
ATR	ENSMUSG00000032409	0.901	-0.151	chr9:95855417-95951644
RAD17	ENSMUSG00000021635	0.892	-0.164	chr13:100617163-100651059
ATM	ENSMUSG00000034218	0.827	-0.274	chr9:53437121-53536671
CHK2	ENSMUSG00000029521	1.048	0.068	chr5:110840016-110874133
XRCC4	ENSMUSG00000021615	0.629	-0.669	chr13:89848913-90089608
XRCC5	ENSMUSG00000026187	0.689	-0.538	chr1:72307420-72394953

XRCC6	ENSMUSG00000022471	0.653	-0.614	chr15:82016368-82040084
PRKDC	ENSMUSG00000022672	0.932	-0.101	chr16:15637865-15842239
LIG4	ENSMUSG00000049717	0.652	-0.616	chr8:9970019-9976323

Supplementary Figure 1

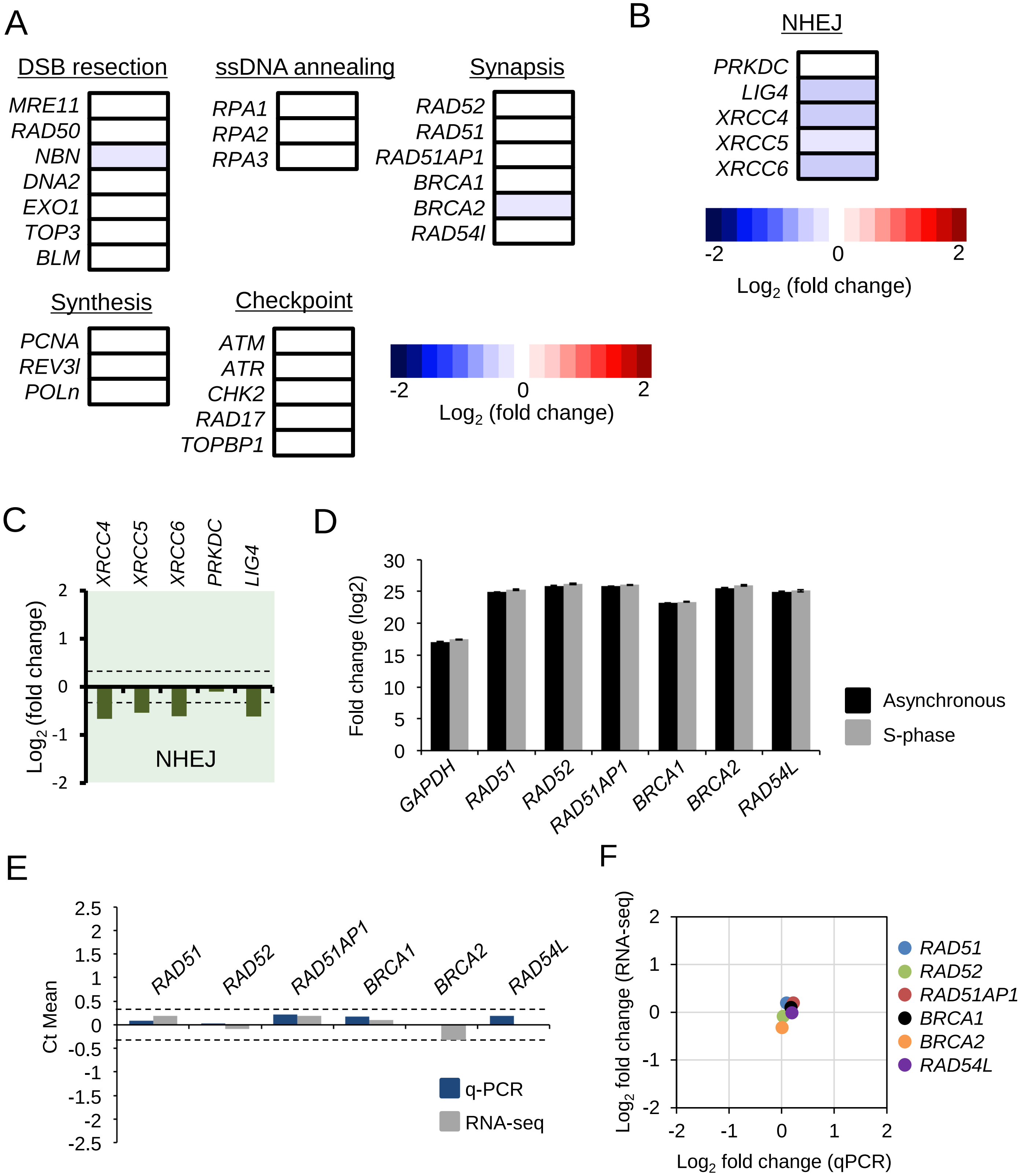

Supplementary Figure 2

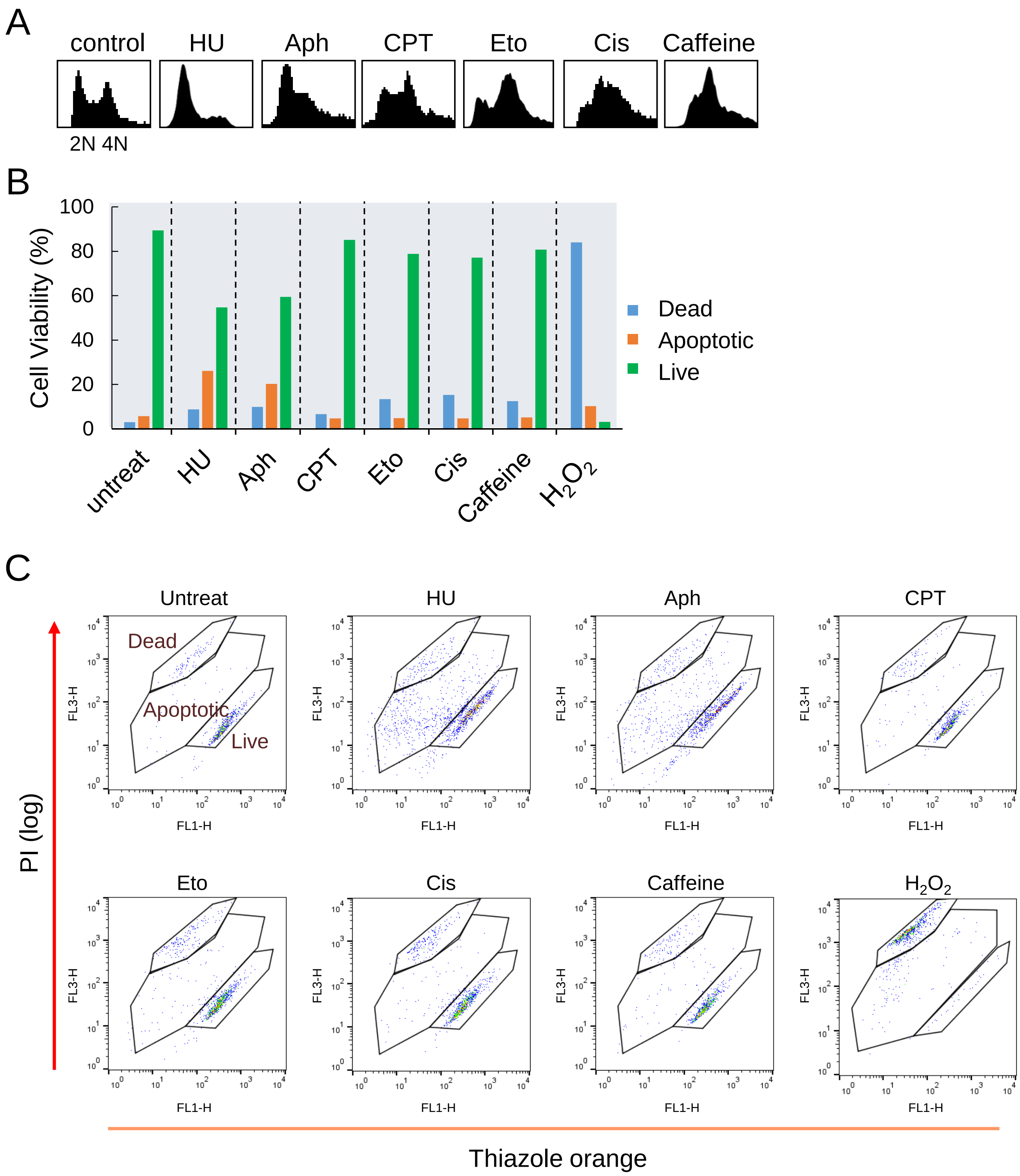

Supplementary Figure 3

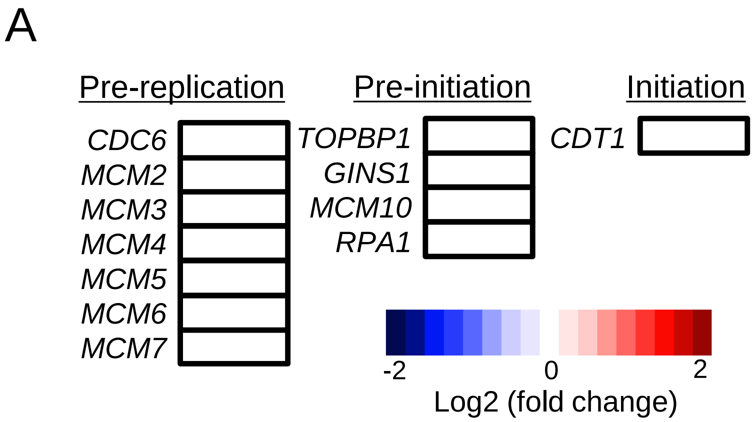

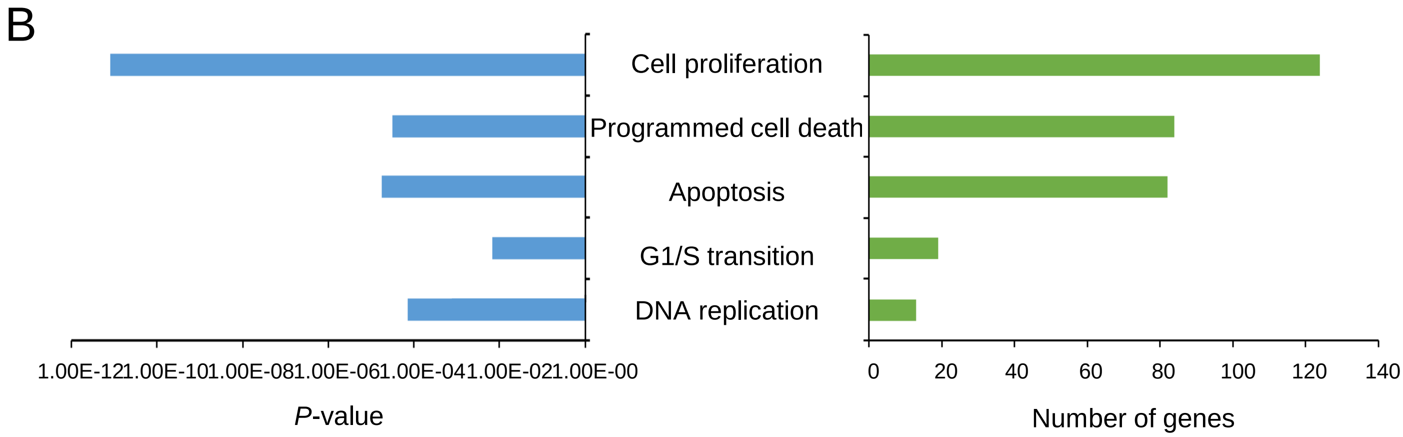

Supplementary Figure 4

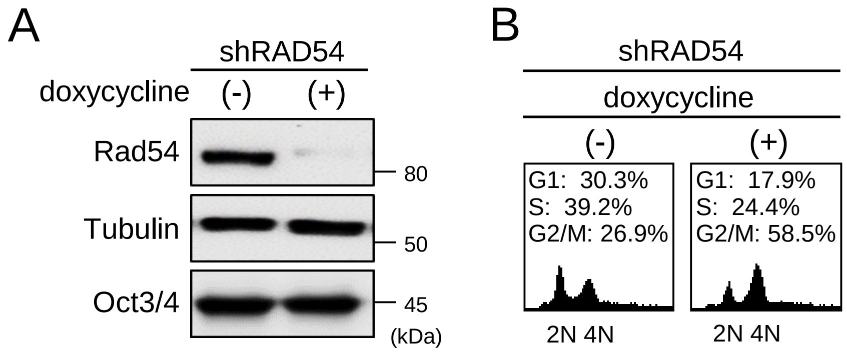

Supplementary Figure 5

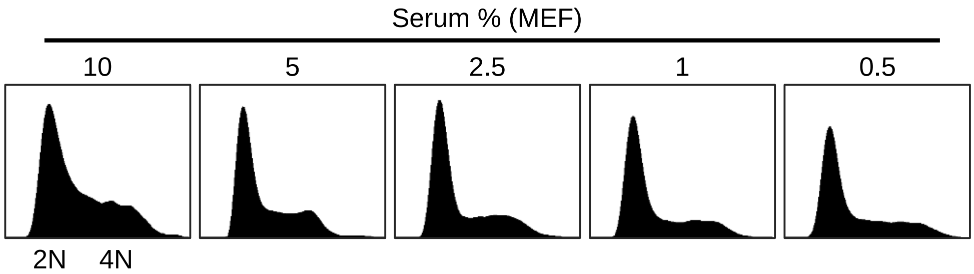

Supplementary Figure 6

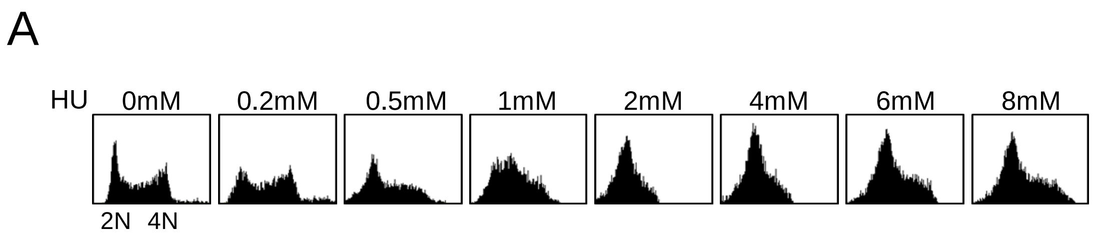

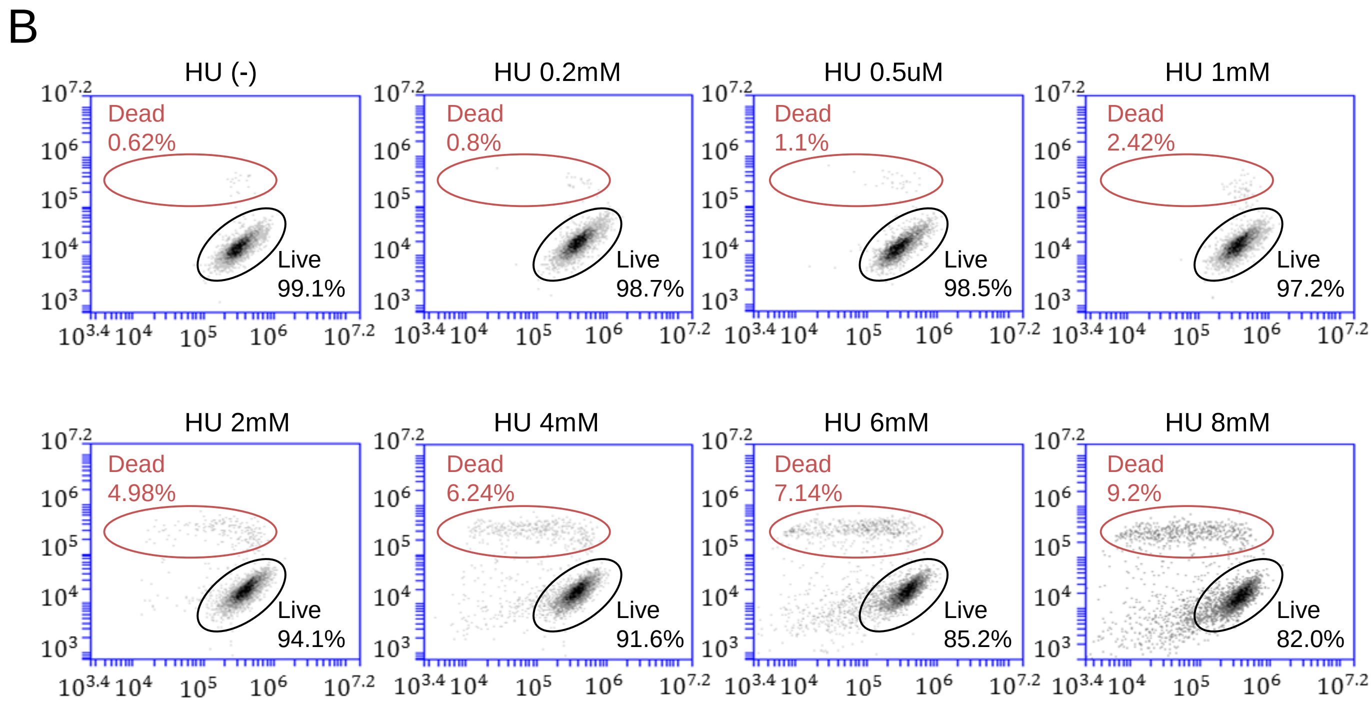

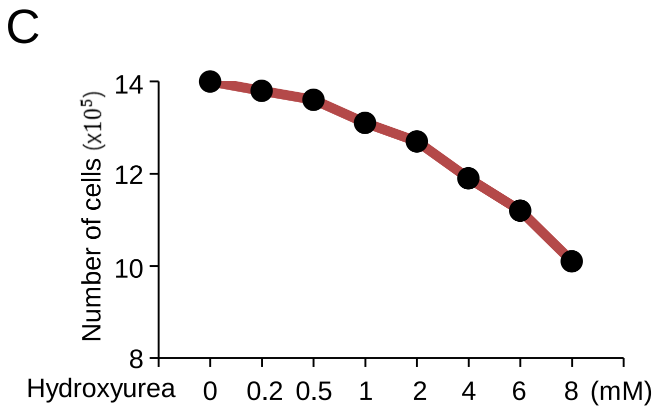

Supplementary Figure 7

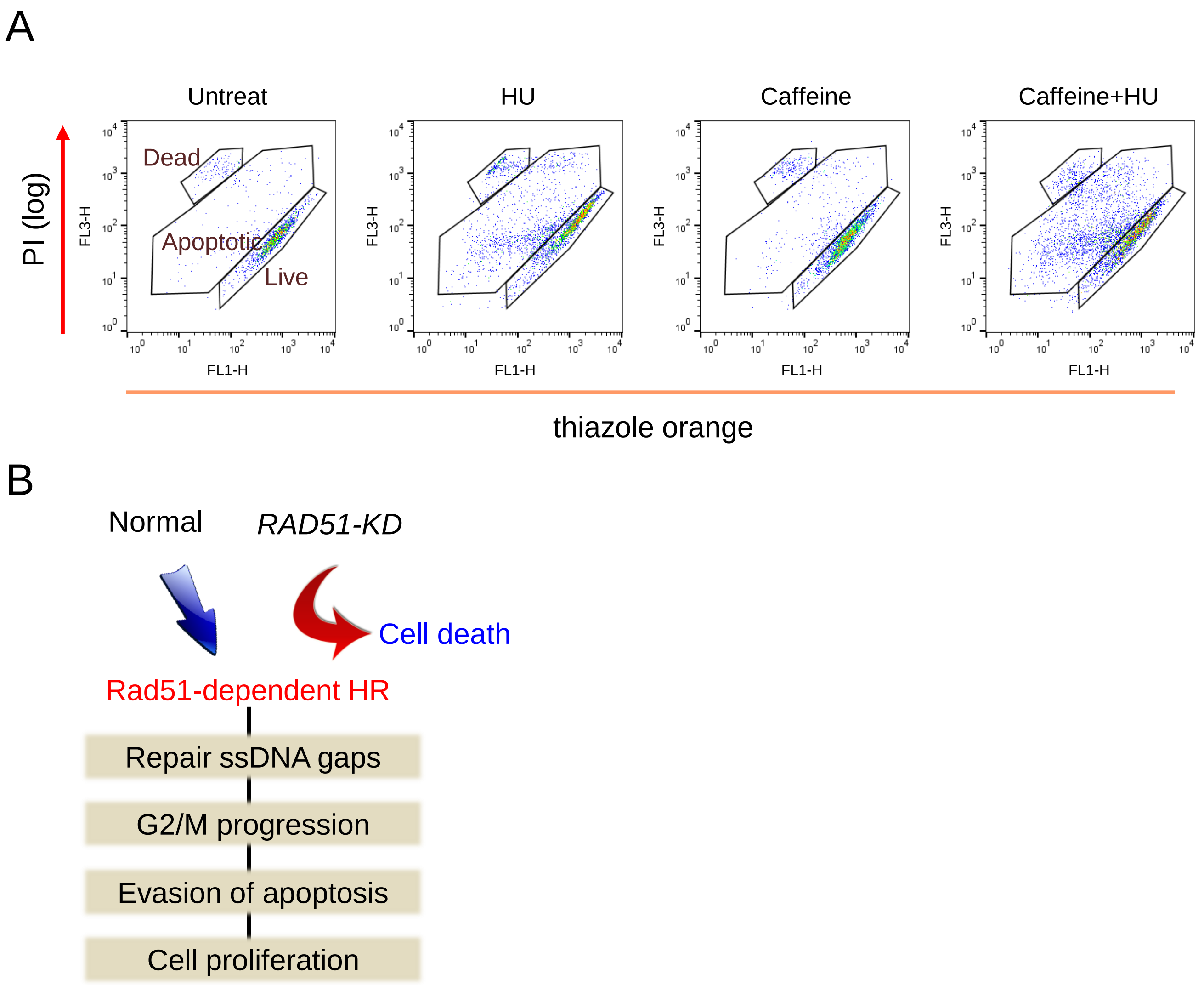

Supplementary Figure 8

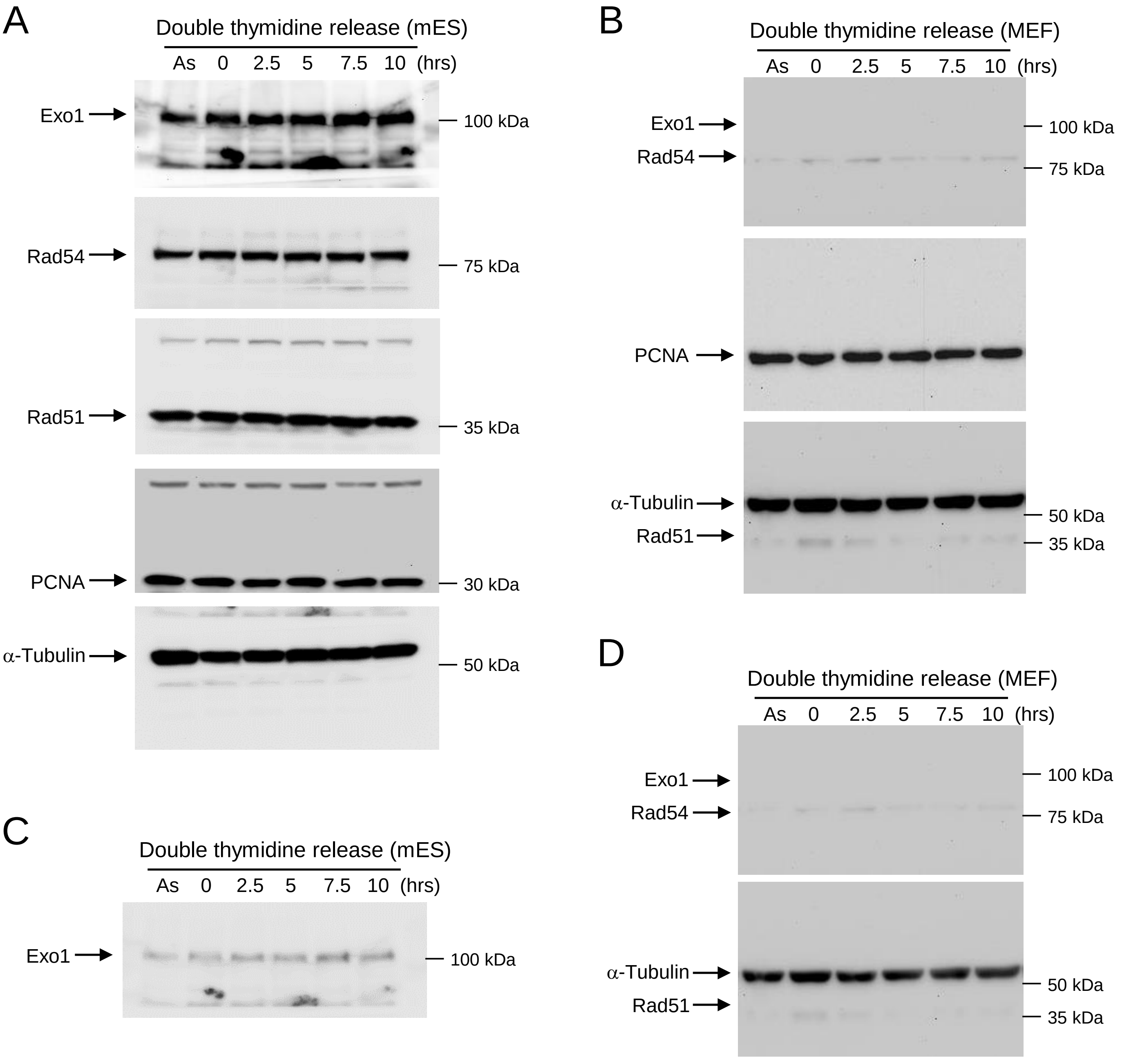

Supplementary Figure 9

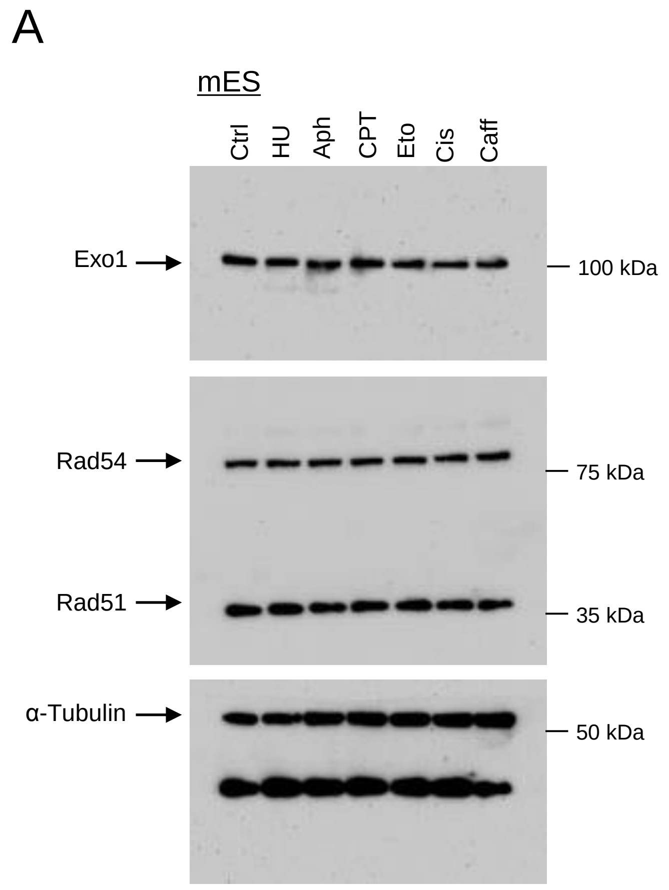

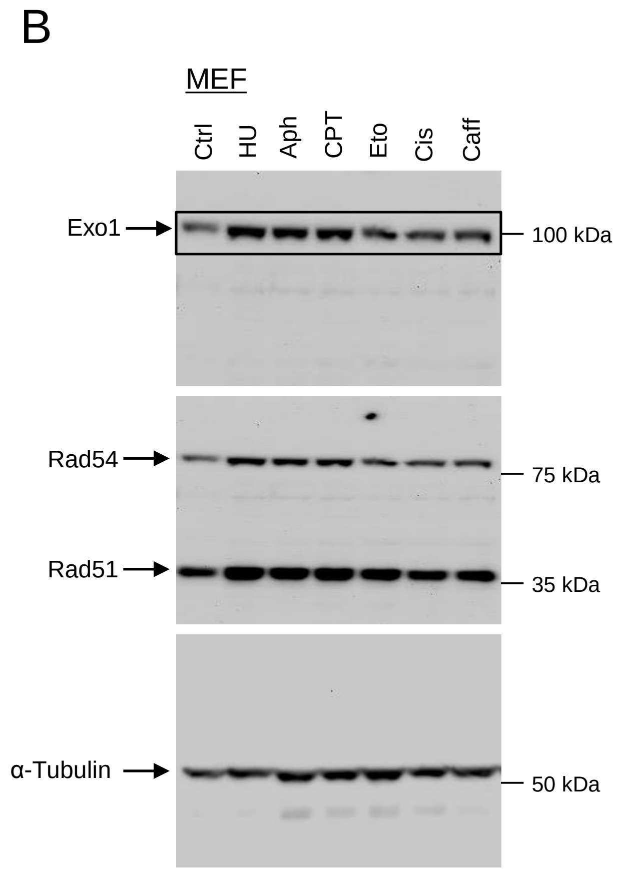

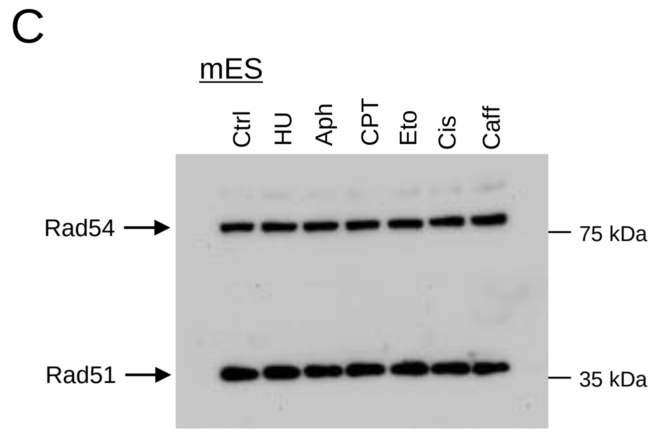

Supplementary Figure 10

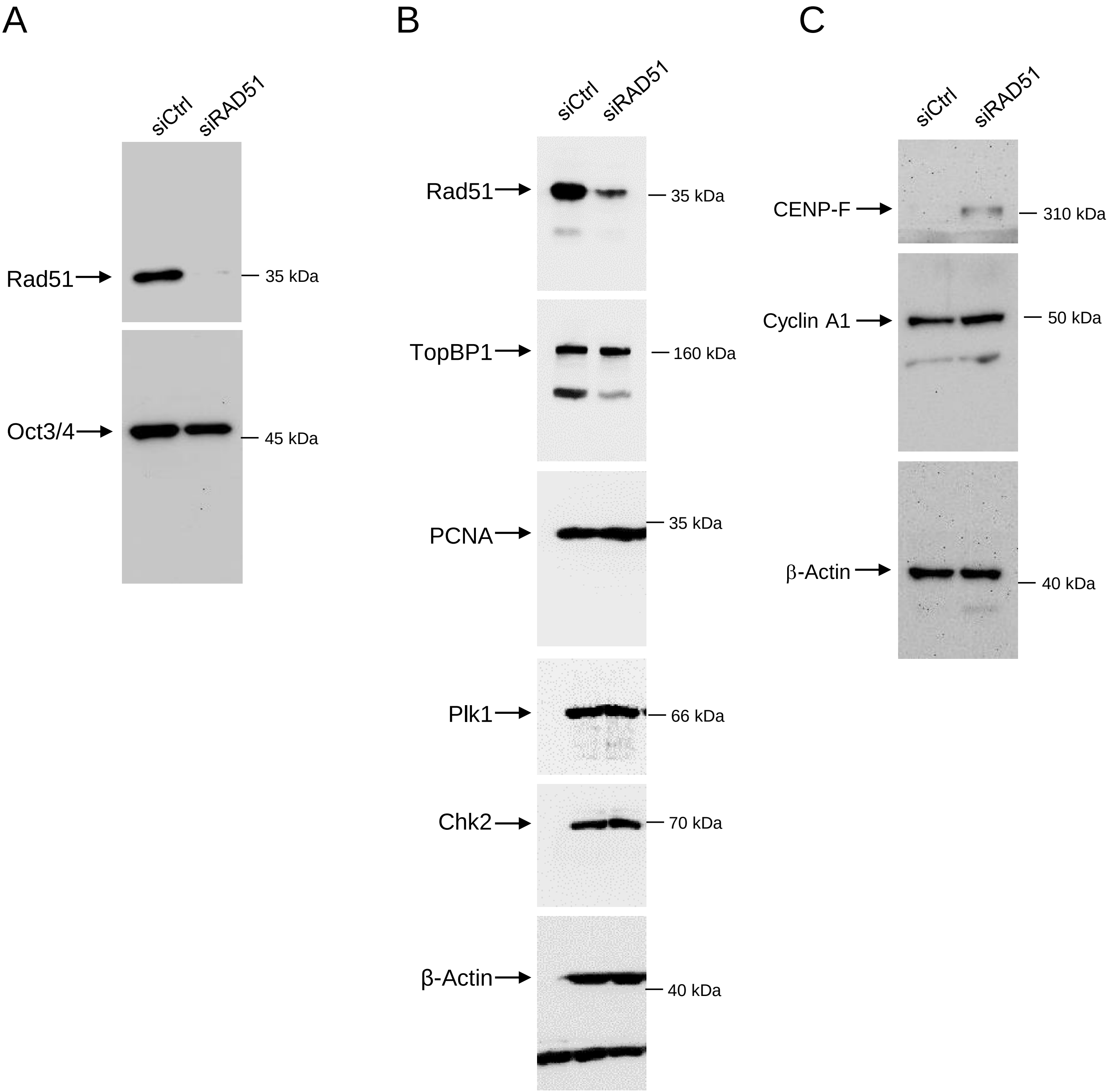

Supplementary Figure 11

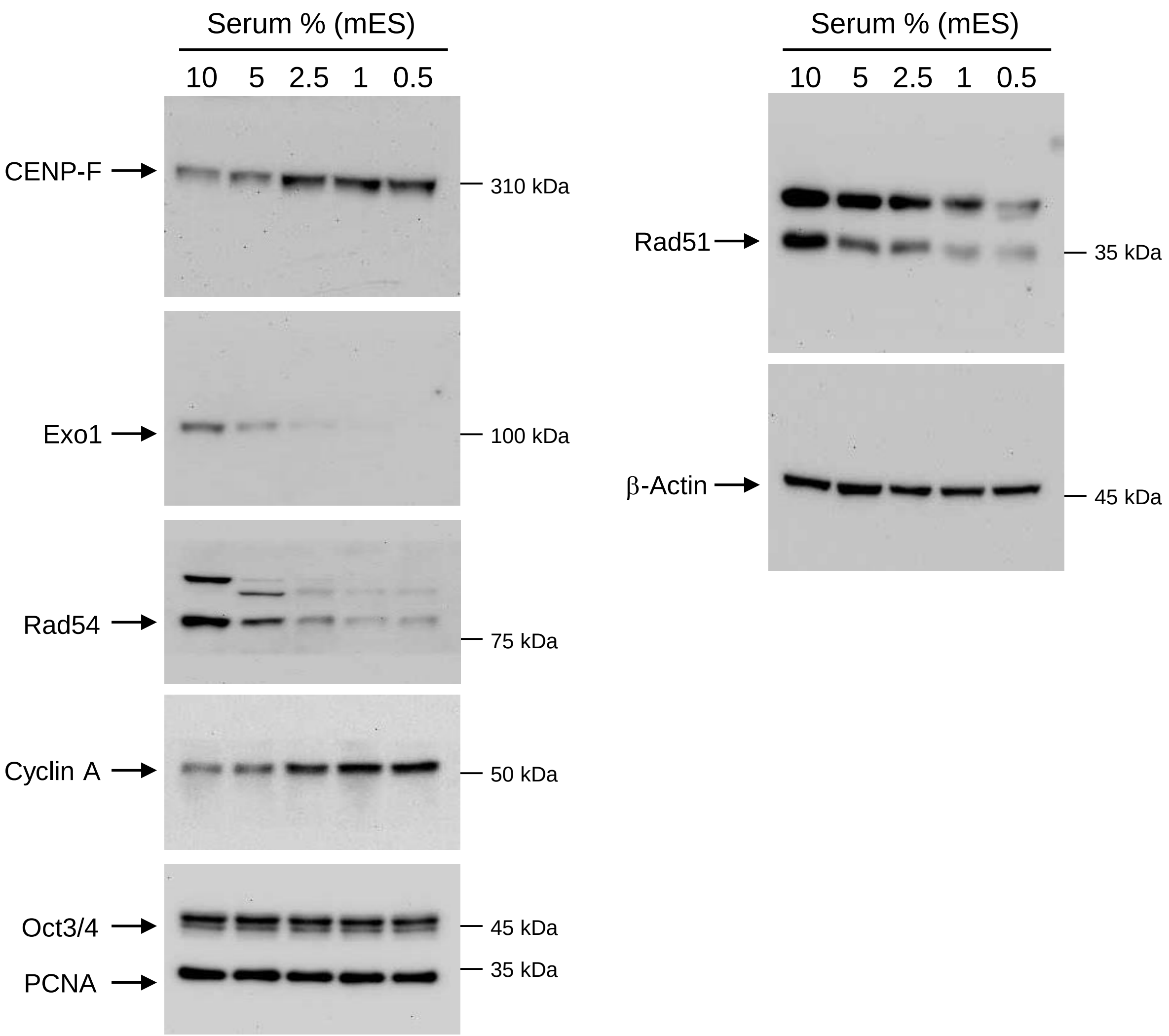

Supplementary Figure 12

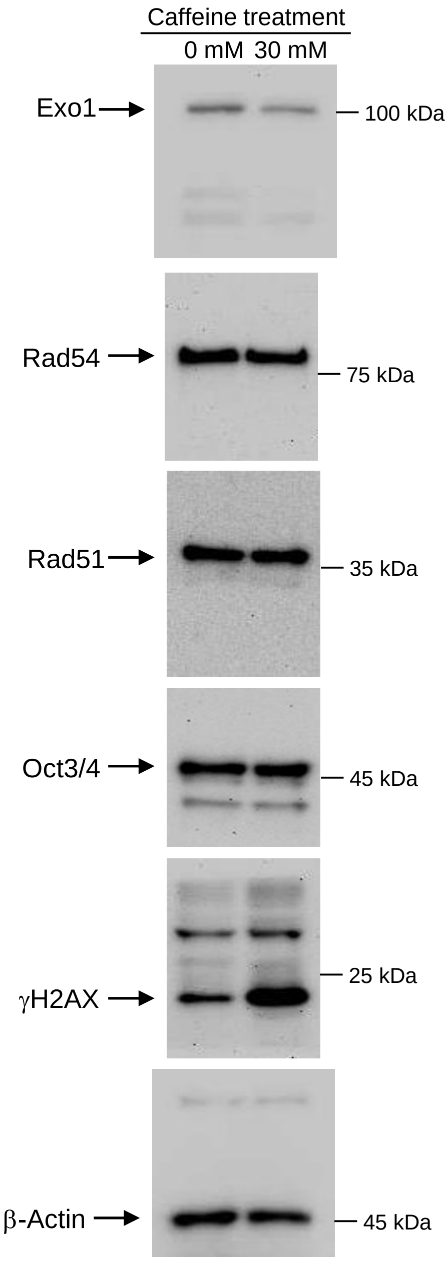

Supplement: Supplementary file 1 — Supplementary information [file 41598_2017_11951_MOESM1_ESM.pdf]
